# Combined casein kinase II and MAPK signalling inhibition as a therapeutic strategy for metastatic colorectal cancer

**DOI:** 10.1186/s40164-026-00855-4

**Published:** 2026-11-05

**Authors:** Mattia Marinucci, Federico Donà, Gina F. Boot, Cinzia Esposito, Elenia Toccafondi, Daniela Liberati, Andrej Benjak, Lara Zaidi, Melania Lazzari, Giulia Milardi, Karolina Guja-Jarosz, Miriam Cieri, Huiche Feng, Mairene Coto-Llerena, Raoul A. Droeser, Savas D. Soysal, Otto Kollmar, Guido Torzilli, Antonino Spinelli, Luigi M. Terracciano, Charlotte K. Y. Ng, Salvatore Piscuoglio

**Affiliations:** 1 Department of Biomedicine, University Hospital Basel, University of Basel, Basel, Switzerland; 2 Molecular Cancer Research, Center for Molecular Medicine, University Medical Center Utrecht, Heidelberglaan 100, 3584 CG Utrecht, The Netherlands; 3 IRCCS Humanitas Research Hospital, Via Manzoni 56, Rozzano, Milan, 20089 Italy; 4 Institute of Medical Genetics and Pathology, University Hospital Basel, University of Basel, Basel, Switzerland; 5 Department for BioMedical Research (DBMR), University of Bern, Bern, Switzerland; 6 Department of Biomedical Sciences, Humanitas University, Via Rita Levi Montalcini 4, 20072 Pieve Emanuele, Milan, Italy; 7 Faculty of Medicine, University of Basel, Klingelbergstrasse 61, Basel, 4056 Switzerland; 8 Department of Visceral Surgery, Clarunis, University Centre for Gastrointestinal and Liver Diseases, St. Clara Hospital and University Hospital Basel, Basel, Switzerland; 9 Kantonsspital Baselland, Liestal, 4410 Switzerland; 10 Oncode Institute, Utrecht, The Netherlands

**Keywords:** Patient-derived organoids, (Phospho)proteomic analysis, Precision medicine, Colorectal cancer liver metastasis, Casein kinase II, Ex vivo drug screening, MAPK signalling

## Abstract

**Background:**

Metastatic colorectal cancer (mCRC) remains a significant clinical challenge due to the emergence of drug resistance, highlighting the need for new therapeutic strategies. Aberrant regulation of the proteome drives cancer progression, and dissecting these alterations offers opportunities for targeted intervention. We aimed to identify candidate kinase dependencies in mCRC and to test a rational combination strategy in patient-derived models.

**Methods:**

We performed proteomic and phosphoproteomic profiling of 10 mCRC tissues, their matched patient-derived organoids (PDOs) and normal colon mucosa, integrated with whole-exome sequencing. Kinase activities were inferred by kinase-substrate enrichment analysis. Silmitasertib-mediated casein kinase II (CKII) inhibition, alone and combined with the MEK inhibitor trametinib, was evaluated by ex vivo drug screening, western blot, flow cytometry and drug-interaction analysis across the PDO cohort and in normal colon PDOs.

**Results:**

PDOs recapitulated the molecular features of matched tumours. Both tissues and PDOs showed consistently elevated CKII activity. Silmitasertib exerted heterogeneous antitumour activity, and resistance was associated with MAPK and Akt-mTOR signalling activity. Combining silmitasertib with trametinib produced an additive reduction of both resistance-associated pathways with little effect on the viability of normal colon PDOs. The combination also significantly reduced CKII activity, the abundance of active β-catenin and the colorectal cancer stem cell pool.

**Conclusions:**

Phosphoproteomic profiling identified CKII as a therapeutic vulnerability in mCRC and provided a rationale for combining silmitasertib with MEK inhibition, offering a candidate combination strategy for mCRC.

**Supplementary Information:**

The online version contains supplementary material available at https://doi.org/10.1186/s40164-026-00855-4.

## Background

Colorectal cancer (CRC) is the third most commonly diagnosed cancer and the second leading cause of cancer deaths worldwide [1]. Metastasis is the primary cause of mortality in CRC, with the liver being the most frequent site for distant metastases [2]. Approximately 20% of CRC patients present with metastases at diagnosis, while up to 60% of patients develop distant metastases within 5 years after the first therapeutic intervention [3]. The median overall survival for metastatic CRC (mCRC) patients with the current standardised therapeutic approaches is around 30 months [4]. Following first- and second-line therapies, the challenge lies in selecting the most appropriate therapeutic options in the third- or fourth-line settings. Current options include TAS-102, regorafenib, or rechallenge with previous chemotherapy, with or without targeted therapy [5]. However, the frequent development of drug resistance remains a significant clinical challenge. Ongoing efforts are focused on testing novel combination therapies that offer additive/synergistic efficacy and, ideally, non-overlapping toxicity [5].

A powerful model that helps guide personalised medicine through ex vivo evaluation of anticancer drug responses is represented by patient-derived organoids (PDOs) [6], a complex model able to recapitulate tumour tissue heterogeneity and architecture. Indeed, several studies have validated the accuracy and sensitivity of PDOs in predicting treatment response, especially in the context of CRC [7]. More recently, large-scale PDO biobanks from CRC liver metastases have further established their value as a resource for functional precision oncology and patient stratification [8]. Yet, identifying conserved molecular vulnerabilities that can guide rational combination therapies in mCRC remains an open challenge.

The aberrant regulation of (phospho)proteomic networks is implicated in cancer development and progression, and advances in mass spectrometry-based technologies, together with the integration of genomic, epigenomic and transcriptomic data, offer an opportunity to unveil cancer vulnerabilities and guide treatment strategies [9, 10].

Drug discovery is driven by the identification of new molecules with beneficial pharmacological activity. However, the success in bringing new molecules with therapeutic potential into the clinic is becoming more and more difficult; thus, combining two or more agents may have multiple favourable impacts in pharmacotherapy [11]. In the present work, we evaluated the (phospho)proteomic features of a cohort of 10 mCRC patients and found that both CRC tissues and PDOs exhibited high casein kinase II (CKII)-related signalling, along with Akt-mTOR and cell cycle-related pathways. Finally, we assessed the efficacy of different single and combined treatments on our ex vivo PDO models to provide a proof of concept for phosphoproteomic data-based design of a personalised treatment approach for mCRC patients.

## Materials and methods

### Patient cohort and tissue sample processing

Ten human CRC liver metastases and six normal colon tissues were obtained from 16 CRC patients undergoing surgery at the University Centre for Gastrointestinal and Liver Disease (Clarunis), Basel, Switzerland. Written informed consent was obtained from all patients. The study was performed in accordance with the Helsinki Declaration and approved by the ethics committee (Ethics Committee of Basel, EKBB, no. 2019-02118). Clinical data were collected retrospectively (Table 1). Tissues were collected after resection and processed within 2 h; mCRC specimens were washed in cold PBS plus primocin, and small pieces were snap-frozen in liquid nitrogen for molecular profiling or processed for PDO generation. An independent validation cohort of six CRC liver metastases was collected at both centres: three at IRCCS Humanitas Research Hospital, Rozzano, Milan, Italy (P563, P581 and P687), under authorisation no. 3631 of the Humanitas Ethics Committee, and three at Clarunis, Basel (P145, P237 and P405), under approval no. 2019-02118. Two primary colorectal tumours and the normal colon mucosa used to establish the PDO lines P134 and P135 were obtained at Humanitas under the same authorisation, whereas the normal colon PDO line P645 was established from mucosa collected at Clarunis. Written informed consent was obtained from all patients (Table 1). Further details are in Additional file 1.

### Cell lines maintenance

CRC cell lines HT-29, CaCo2 and HCT-116 were obtained from ATCC, authenticated by short tandem repeat profiling, and routinely tested for mycoplasma contamination by PCR [12]. Cells were cultured in DMEM supplemented with 10% foetal bovine serum, non-essential amino acids and penicillin/streptomycin (all GIBCO), maintained at 37 °C in 5% CO₂ and used while exponentially growing. Further details are in Additional file 1.

### Patient-derived organoid generation and passaging

PDOs were generated from CRC liver metastases, primary colorectal tumours and normal colon tissues according to a previously published protocol [12], using a CRC-specific medium and, for normal colon PDOs, IntestiCult media (Stemcell Technologies); normal colon PDOs were used in the differentiated state for all experiments. Further details are in Additional file 1.

### Immunohistochemistry

Tissues and matched PDOs were fixed in 4% paraformaldehyde (PFA). PDOs were pre-embedded in HistoGel (Thermo Fisher Scientific, #12006679), and both tissues and PDOs were then embedded in paraffin and cut into 3.5 μm-thick sections. Haematoxylin and eosin (H&E) staining was performed according to standard protocols as described previously [13]. See Additional file 1 for details.

### Nucleic acid extraction and RT-qPCR

Genomic DNA and total RNA were co-extracted using the Quick-DNA/RNA kit (Zymo Research, #D7003) following the manufacturer’s instructions and quantified with Qubit assays (Thermo Fisher). For gene expression analysis, RNA was reverse-transcribed and Wnt target/stem-cell markers were measured by RT-qPCR with PowerUp SYBR Green (Applied Biosystems), using the primers listed in Additional file 2. Expression was normalised to the housekeeping gene RPLP0 and, for each marker, to control samples. Experiments were performed at least three times in triplicate (mean ± SD). Further details are in Additional file 1.

### Whole-exome sequencing

According to the availability of biological material, whole-exome sequencing was performed on 8 CRC tissues and matched PDOs, as well as peripheral blood samples as germline controls (except for P17 and P36). Extracted DNA was sent to the Beijing Genome Institute for whole-exome sequencing.

DNA libraries were prepared starting from at least 500 ng of DNA. DNA was fragmented, size-selected (200–300 bp), end-repaired, and ligated with adapters. Libraries were hybridised using the Agilent SureSelect Human All Exon V6 kit for enrichment before sequencing with 150 bp paired-end reads on the DNBSEQ platform. Tumour/PDO samples and non-tumoural samples were sequenced to median depths of 76 (range 49–86) and 44 (range 35–46), respectively. See Additional file 1 for details.

### Proteomic and phosphoproteomic profiling and analysis

**Fig. 1:**
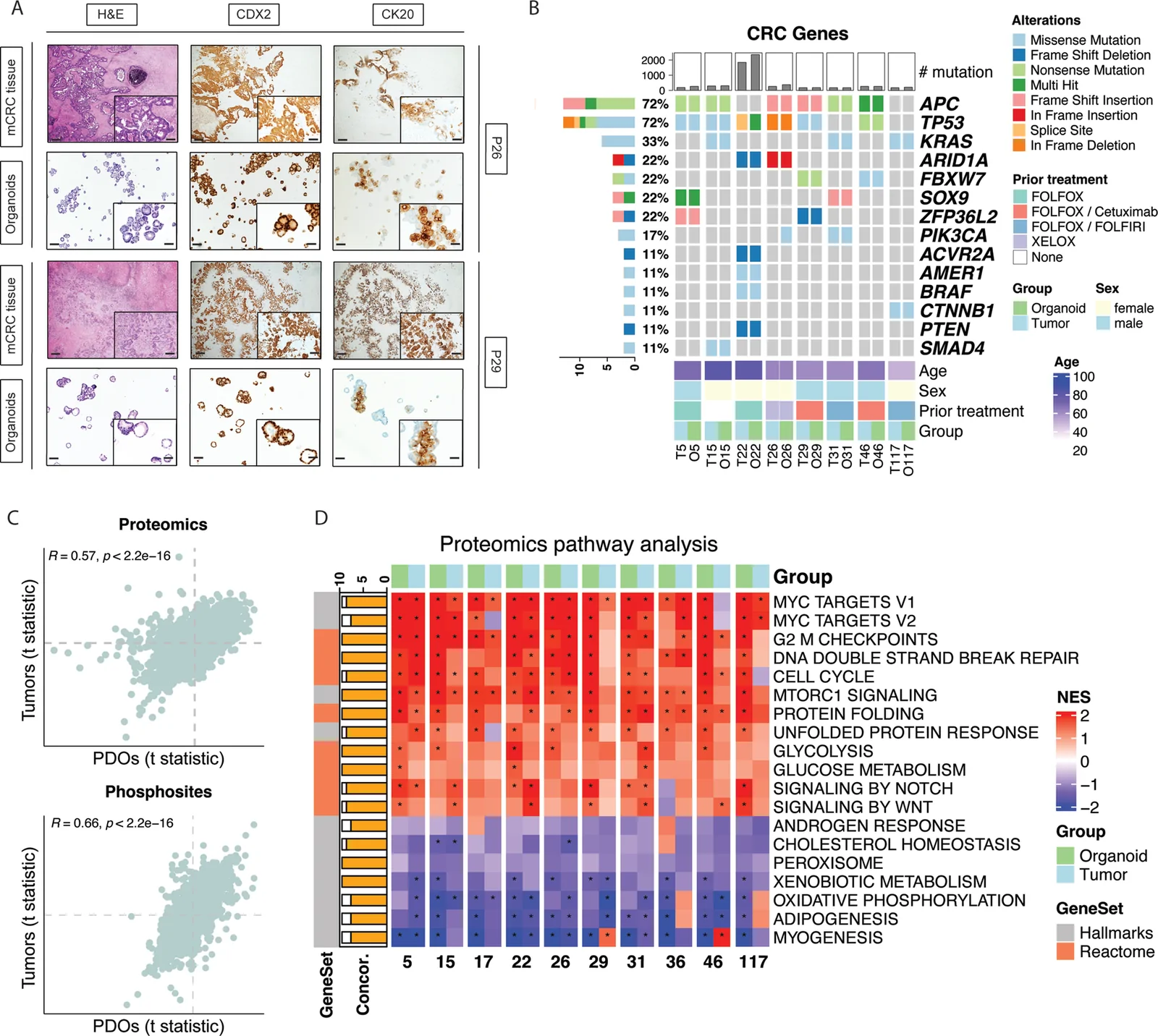
Establishment and characterisation of patient-derived organoids from metastatic colorectal cancer tissues. (**A**) Representative images of H&E and IHC staining for CDX2 and CK20 in two mCRC tumour tissues and matched PDOs. Insets highlight detailed regions of interest. For tissue pictures, scale bars indicate 100 µm and 25 µm for lower and higher magnifications, respectively. For PDO pictures, scale bars are 50 µm and 10 µm for lower and higher magnifications, respectively. (**B**) Oncoprint displaying somatic mutations in CRC driver genes across 8 mCRC tumour tissues and matched PDOs (except P17 and P36). The colour bars represent the types of genomic alterations, as specified in the legend. The top barplot depicts the number of total mutations in each sample. The bar plot on the left illustrates the fraction of samples with mutations in each gene. Clinical annotations are shown below. Prior treatment abbreviations: FOLFOX (folinic acid, fluorouracil and oxaliplatin), FOLFIRI (folinic acid, fluorouracil and irinotecan), XELOX (capecitabine and oxaliplatin), alone or in combination with cetuximab; None, no prior treatment. (**C**) Scatter plots comparing the t-statistics of proteomic and phosphosite data between tumour samples and PDOs. Spearman’s correlation coefficients (R) and p-values are indicated for each comparison. (**D**) Heatmap of pathway enrichment analysis (normalised enrichment score, NES) for individual PDOs and tumour tissues across Hallmark and Reactome gene sets. The source collection of each pathway is indicated by the ‘GeneSet’ colour bar on the left (grey, Hallmark; orange, Reactome); enrichment analysis was performed against these complementary MSigDB collections, which capture broad biological states (Hallmark) and finer pathway-level processes (Reactome), respectively. Black asterisks indicate a significant enrichment (adjusted *p* < 0.05). Grouping information of samples (T=Tumour, O=Organoid) is shown. Enrichment concordance (Concor., orange bars on the left) indicates, for each pathway, the number of matched tumour/PDO pairs showing the same direction of enrichment (scale 0–10)

Proteomic and phosphoproteomic profiling was performed on 10 CRC tissues and matched PDOs, as well as 6 normal colon tissues, using three pieces per resection for tissues and three biological replicates per PDO line. Following protein extraction, digestion and Fe(III)-IMAC phosphopeptide enrichment, peptides were analysed by liquid chromatography-tandem mass spectrometry (LC-MS/MS) on an Orbitrap Fusion Lumos mass spectrometer (Thermo Fisher Scientific); precursor intensities were quantified with Progenesis QI and SafeQuant. Differential abundance (proDA), pathway enrichment (gene set enrichment analysis, GSEA) and kinase-substrate enrichment (KSEA) analyses were performed as detailed in Additional file 1. Comparisons of numerical variables were performed using the Mann–Whitney U test, Kruskal–Wallis test or unpaired two-tailed t-test for unpaired data, the Wilcoxon signed-rank test or paired two-tailed t-test for paired data, and one-way or two-way ANOVA with Tukey’s or Šídák’s post-hoc test for multiple-group comparisons. Genome-wide comparisons of proteomic and phosphoproteomic t-statistics between tumours and PDOs (Fig. 1C) were assessed using Spearman’s rank correlation, whereas all other correlations, including those between kinase or pathway activity scores and drug response, were assessed using Pearson’s correlation coefficient. All tests were two-sided, with *p* < 0.05 considered significant; where appropriate, p-values were corrected for multiple testing using the Benjamini-Hochberg method or, for the two planned comparisons in Fig. 5J, the Holm method. Statistical analyses were performed in GraphPad Prism 10, and the specific test used is indicated in each figure legend.

### Drug treatment on CRC PDO and cell lines

All drugs used are listed in Additional file 2. For cell lines (HT-29, CaCo2, HCT-116), cells were seeded in 384-well plates, treated after 24 h using a D300e Digital Dispenser (Tecan), and cell viability was measured after 72 h with CellTiter-Glo (Promega). CRC PDOs were seeded in Matrigel domes in 96-well plates and, once organoids were visible, treated by replacing the medium with drug-containing medium; viability was measured after 4 days with CellTiter-Glo 3D (Promega). Readings were taken on a Varioskan LUX reader (Thermo Scientific) and normalised to vehicle (0.1% DMSO). Viability curves were fitted by nonlinear regression in Prism 10 (GraphPad). All experiments were performed at least three times in triplicate (mean ± SD). Drug interactions were computed with SynergyFinder 3.0 [14] using the HSA, Bliss, Loewe and ZIP models and classified as synergistic, additive or antagonistic as detailed in Additional file 1.

### Immunoblotting

Total proteins were extracted from tissues and PDOs in Co-IP lysis buffer supplemented with protease and phosphatase inhibitors (Roche), separated by SDS-PAGE [15] and transferred to nitrocellulose membranes. Membranes were probed with primary antibodies overnight at 4 °C and with fluorescent secondary antibodies (LI-COR Biosciences); primary and secondary antibodies are listed in Additional file 2. Blots were scanned on an Odyssey Infrared Imaging System (LI-COR) and band intensity was quantified with ImageJ, normalising the protein of interest to a β-actin or GAPDH loading control. Protein activity was calculated as the ratio of phosphorylated to total protein, mirroring the approach used for the phosphoproteomic dataset. Further details are in Additional file 1.

### Flow cytometry analysis

Cell cycle was assessed by DAPI-based detection of DNA content. PDOs were treated with silmitasertib (10 µM) or vehicle (0.1% DMSO) for 24 h, dissociated to single cells, fixed in 70% ethanol, stained with DAPI and analysed by flow cytometry (CytoFLEX, Beckman Coulter). For cancer stem cell markers, PDOs treated for 24 h with vehicle, silmitasertib (10 µM), trametinib (30 nM) or their combination were dissociated and stained with DAPI for live/dead exclusion, APC-conjugated anti-CD133 and AF488-conjugated anti-CD44 (Additional file 2) before acquisition on a CytoFLEX cytometer. Data were analysed using FlowJo 10.10 (BD). Further details in Additional file 1.

### Caspase-3/7 activity

Caspase-3/7 activity was measured in PDOs treated with vehicle, silmitasertib, trametinib or their combination using the Caspase-Glo 3/7 3D assay (Promega) on a Varioskan reader (Thermo Fisher), normalised to vehicle (0.1% DMSO). Experiments were performed at least three times in triplicate (mean ± SD). Further details in Additional file 1.

### Use of AI-assisted tools

During the revision, an AI assistant (Claude, Anthropic) was used to support language editing and harmonisation. All outputs were reviewed and validated by the authors, who take full responsibility for the content.

## Results

### Establishment and characterisation of patient-derived organoids from metastatic colorectal cancer tissues

To establish a living biobank of PDOs from mCRC for drug screening, we collected surgically resected tissue specimens from the liver metastasis of 10 CRC patients and generated PDO cultures (Table 1). All tumours exhibited typical adenocarcinoma features. Half of the patients were male, with a mean age of 64.5 years (range 50–81 years). Most (9/10) had received a first-line standard chemotherapy regimen. Normal colon mucosa samples were collected from six further CRC patients from an unrelated cohort, undergoing resection of their primary tumour, and used as reference for the (phospho)proteomic analyses (mean age 74.3; range 61–84 years).

All mCRC PDOs except P17 showed long-term expansion capacity. Histopathological characterisation revealed that PDOs closely mirrored the adenocarcinoma architecture and cellular morphology of their matched tumours, including glandular structures with varying degrees of differentiation, hyperchromasia and a high nucleus-to-cytoplasm ratio (Fig. 1A and Additional file 3: Fig. S1A). PDOs also retained the expression patterns for CDX2 and CK20, confirming their colorectal tissue origins (Fig. 1A and S1A), and recapitulated the microsatellite stability status of the matched tissues, with all PDOs being microsatellite stable except for P22, which was indeed negative for MLH1 and PMS2 (Fig. S1B).

Whole-exome sequencing analysis demonstrated that mutations in CRC driver genes were preserved in all sequenced PDOs (Fig. 1B). In our cohort, mutations were highly prevalent in *APC* and *TP53* (72% of samples) and a third of the patients had oncogenic *KRAS* mutations (G12D in P15 and P31, G13D in P117), reflecting population mutation rates [16] (Fig. 1B). To confirm the stability of the genomes over time, the PDOs of three randomly selected patients were further investigated at the stage of short- and long-term passaging, demonstrating the conservation of all driver mutations through passaging (Fig. S2A and S2B).

To identify potential therapeutic targets, we performed proteomic and phosphoproteomic profiling of the mCRC tumour tissues and their matched PDOs, with parallel profiling of a pool of normal colon mucosa tissues as reference, quantifying 4,254 proteins and 6,964 phosphosites for downstream analysis. At the cohort level, overall dysregulation of the proteome and phosphoproteome was conserved between the PDOs and tumour tissues compared to normal colon mucosa (Fig. 1C, Spearman’s *R* = 0.57 and 0.66, respectively). Pathway enrichment analysis of these dysregulated proteomes revealed high concordance of mCRC-relevant pathways in PDOs and tumours, such as upregulation of MYC targets, G2M checkpoints, signalling by WNT and MTORC1 signalling (Fig. 1D). Compared to PDO models, both tumour and colon mucosa tissues showed higher expression of proteins related to stromal and immune-related pathways such as complement cascade and extracellular matrix-receptor interactions, resulting in high immune and stromal scores (Fig. S2C-D). This finding is consistent with the fact that ex vivo PDO models lack both immune and stromal components. Moreover, the tumour-containing samples (tumour tissues and PDOs) had higher expression of DNA replication-related proteins (Fig. S2C). Upon batch-correcting for these systematic differences between sample groups, we observed that the paired tumours and PDOs displayed a high degree of similarity on the proteomic and phosphoproteomic levels (Fig. S2E).

In summary, PDOs accurately mirrored the histological and molecular characteristics of their originating mCRC tumour tissues, despite not retaining immune and stromal components. This molecular characterisation showcases the utility of PDOs as a representative model for mCRC and supports their relevance as preclinical tools in cancer research.

### CKII activity is upregulated in metastatic CRC tissues and PDOs

**Fig. 2:**
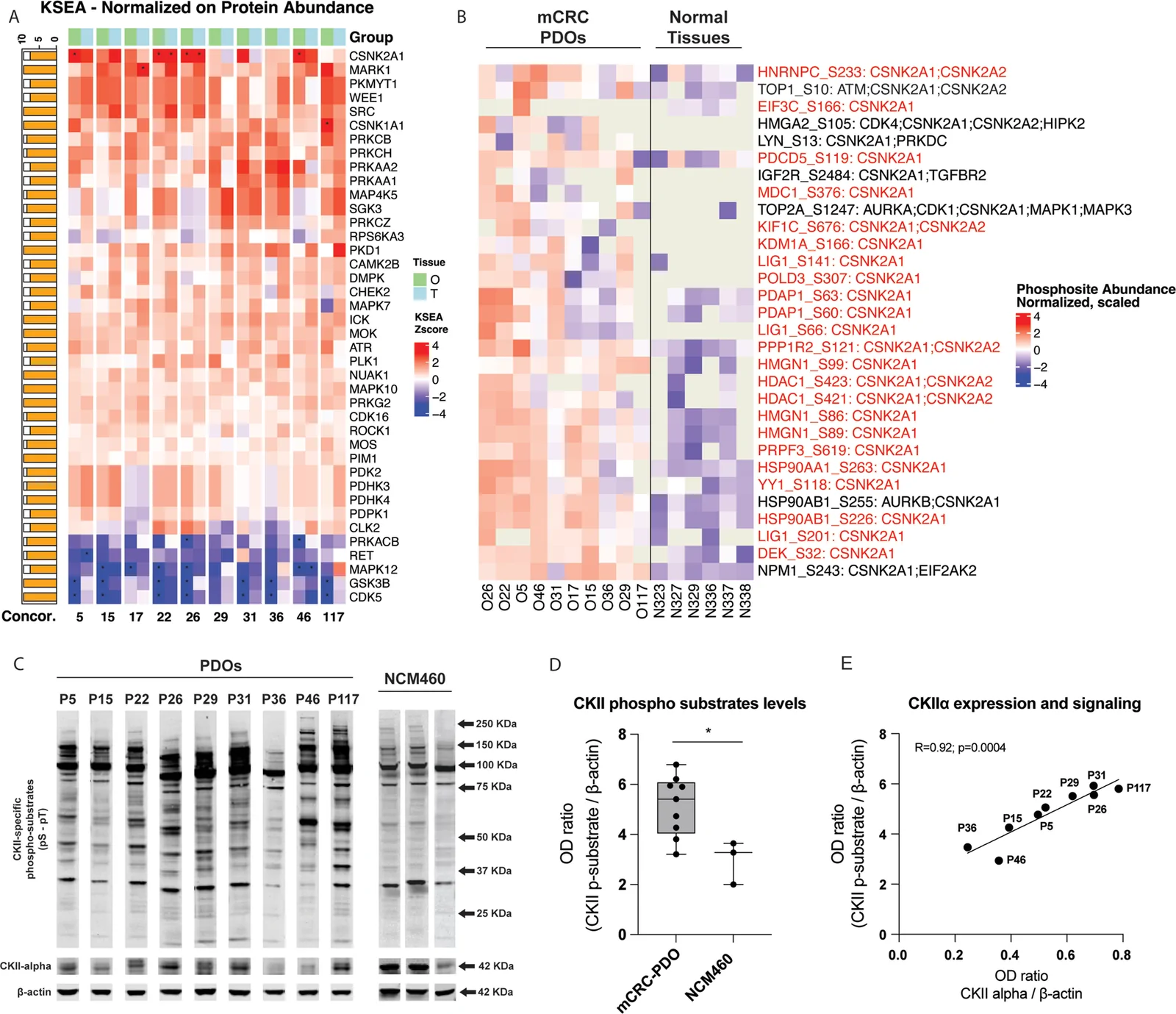
CKII activity is upregulated in metastatic CRC tissues and PDOs. (**A**) KSEA heatmap based on protein abundance normalisation showing kinase activity of the top 40 concordant kinases across PDOs and mCRC tumour samples. Black asterisks indicate significant enrichment (adjusted *p* < 0.05). Grouping information of samples (T=Tumour, O=Organoid) is shown above. Enrichment concordance (Concor., orange bars on the left) indicates, for each kinase, the number of matched tumour/PDO pairs showing the same direction of enrichment (scale 0–10). (**B**) Heatmap depicting unimputed normalised abundance of CKII-specific phosphosubstrates across PDOs and normal tissues. Grey colour represents missing values. Each row represents a phosphosubstrate separated by colons from the respective upstream kinase. The text in red indicates that the phosphosubstrates are phosphorylated exclusively by CKII, while the text in black highlights phosphosubstrates with a known or predicted relationship to other kinases in the dataset. (**C**) Representative western blot images showing CKII-specific phosphosubstrate levels (pSerine/pThreonine), CKIIα expression, and β-actin loading control in mCRC PDOs and the normal colon mucosa cell line NCM460. All lanes were cropped from the same blot. (**D**) Quantification of CKII phosphosubstrate levels normalised to β-actin in mCRC PDOs compared to NCM460. For mCRC PDOs, each dot represents an individual PDO line (*n* = 9); for NCM460, each dot represents an independent biological replicate (*n* = 3). Boxes indicate the median and interquartile range, and whiskers the minimum and maximum. Statistical significance was assessed using Mann-Whitney U test (*p < 0.05). (**E**) Pearson correlation between CKIIα expression and CKII phosphosubstrate levels across PDOs (*n* = 9; *R* = 0.92, *p* = 0.0004)

Recognising the critical role of proteins and kinases in cancer progression and their potential as drug targets, we investigated their putative dysregulated signalling and activity by analysing the proteomic and phosphoproteomic profiles of both mCRC tissues and matched PDOs. In both models, kinase-substrate enrichment analysis (KSEA) of differentially regulated phosphosites identified CSNK2A1 as one of the most active kinase subunits, both with and without normalisation of phosphosubstrate levels to protein abundance, whereas other kinases involved in proliferation pathways, such as cyclin-dependent kinases (CDK1), exhibited enrichment only without normalisation (Fig. 2A and S3A). Similarly, performing a group-wise analysis on the PDO cohort, we found CKII to be among the most active kinases (Fig. S3B), prompting us to further assess its signalling activity.

By investigating the CKII-specific phosphosubstrate pool, our analysis revealed that the majority (77%) of the thirty most differentially phosphorylated CKII substrates were uniquely phosphorylated by CKII catalytic subunits (Fig. 2B), strengthening the reliability of our dataset. We also observed positive correlations with two important protein factors known to regulate and enhance CKII activity, namely CSNK2B [17] and NELFE [18] (Fig. S3C).

To validate our findings, we examined the phosphorylation status of CKII-specific substrates as an indicator of CKII activity in our PDO cohort and the normal colon mucosa cell line NCM460 [19]. We confirmed that the phosphorylation levels of CKII-specific substrates were significantly higher in our PDO models compared to NCM460 (Fig. 2C, D). Interestingly, we also found that a higher phosphorylation of CKII-specific substrates corresponded to higher expression levels of CKIIα subunit (Fig. 2E).

Taken together, our protein and phosphosite characterisation revealed various dysregulated pathways and kinase activities within our mCRC patient cohort and, in particular, consistently pointed to an overactivation of CKII in both metastatic CRC tissues and matched PDOs. These findings prompted us to further explore the anticancer potential of targeting CKII in mCRC patient-derived organoid models.

### Silmitasertib exerts antitumour effects and induces DNA damage and apoptosis in mCRC PDOs

We decided to investigate the antitumour efficacy of targeting CKII and CDK1 kinases, which emerged as the top candidates following normalised and non-normalised kinase activity analysis (Fig. 2A and S3A, respectively). To target CKII and CDK1 pathways, we selected silmitasertib and dinaciclib, respectively. Both are clinical-stage inhibitors that have received FDA orphan drug designation (silmitasertib for cholangiocarcinoma and other rare tumours; dinaciclib for chronic lymphocytic leukaemia) and have been evaluated in clinical trials, including in solid malignancies.

To explore drugs’ dynamic ranges to be used on CRC PDOs treatment, we first screened their efficacy on a small panel of immortalised CRC cell lines bearing different driver mutations and features [20] (Fig. S4A, B).

**Fig. 3:**
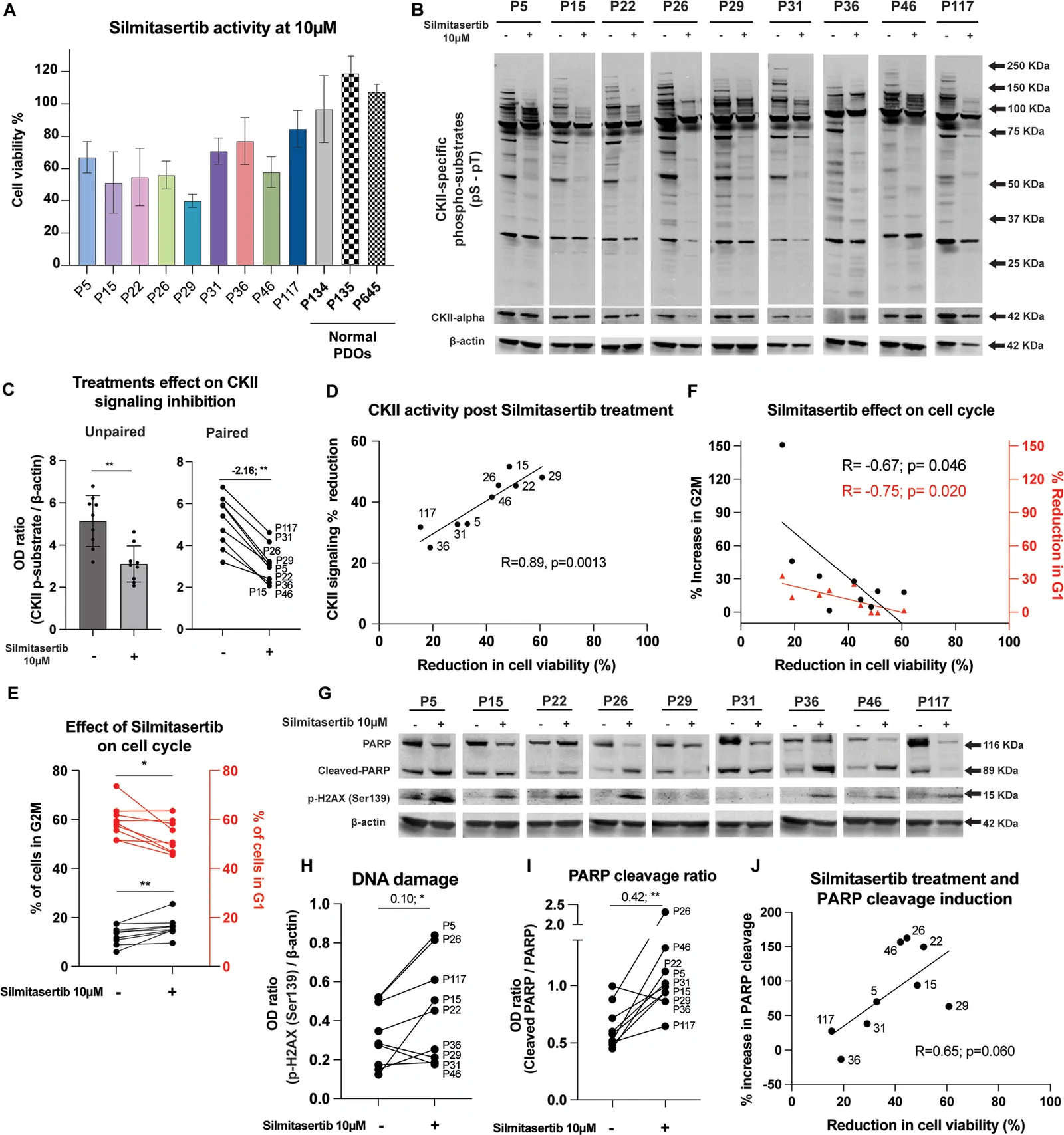
Silmitasertib exerts antitumour effects and induces DNA damage and apoptosis in mCRC PDOs. (**A**) Bar plot showing cell viability of mCRC PDOs and normal colon PDOs following silmitasertib treatment at 10 µM. Data are presented as mean ± SD of at least three independent experiments performed in triplicate. (**B**) Western blot showing CKII-specific phosphosubstrate levels (pSer/pThr motif), CKIIα expression and β-actin loading control in mCRC PDOs treated with silmitasertib 10 µM or DMSO. (**C**) Quantification of CKII phosphosubstrate levels before and after silmitasertib treatment (*n* = 9 PDO lines), shown as unpaired (left, Mann-Whitney U test) and paired (right, Wilcoxon signed-rank test) comparisons. In the unpaired panel, bars represent mean ± SD. Numbers adjacent to significance brackets indicate the median difference. Statistical significance: **p < 0.01. (**D**) Scatter plot showing the correlation between CKII signalling reduction and reduction in cell viability following silmitasertib treatment across mCRC PDOs (*n* = 9; *R* = 0.89, *p* = 0.0013). Pearson’s correlation coefficient (R) and the corresponding p-value are indicated. (**E**) Line graph showing the percentage of cells in G2/M (black) and G1 (red) phases before and after silmitasertib treatment across mCRC PDOs (*n* = 9). Statistical significance was assessed using the paired Wilcoxon signed-rank test (**p < 0.01, *p < 0.05). (**F**) Scatter plots showing correlations between silmitasertib-induced changes in G2/M (black) and G1 (red) cell fractions and reduction in cell viability across mCRC PDOs (*n* = 9). The Pearson correlation coefficients (*R* = −0.67, *p* = 0.046 for G2/M; *R* = −0.75, *p* = 0.020 for G1) are provided. (**G**) Western blot showing PARP, cleaved-PARP, γ-H2AX (p-H2AX Ser139) and β-actin in mCRC PDOs treated with silmitasertib 10 µM or DMSO. (**H**) Quantification of DNA damage via γ-H2AX to β-actin ratio and (I) PARP cleavage ratio (cleaved PARP/total PARP) in PDOs (*n* = 9 PDO lines) following 24 h treatment with silmitasertib (10 µM). Statistical significance is determined by the Wilcoxon signed-rank test (*p < 0.05; **p < 0.01), while numbers indicate the median difference. (**J**) Scatter plot showing the correlation between silmitasertib-induced PARP cleavage and reduction in cell viability across mCRC PDOs (*n* = 9; *R* = 0.65, *p* = 0.060). Pearson’s correlation coefficient (R) and the corresponding p-value are indicated

While silmitasertib treatment exhibited significant antitumour activity on mCRC PDOs at a concentration of 30 µM (Fig. S4C), patient-specific resistance/sensitivity was noticed at 10 µM concentration (Fig. 3A). Interestingly, normal colon PDOs (Fig. S5A) were not sensitive to 10 µM and were slightly sensitive to 30 µM (Fig. 3A and Fig. S4C), suggesting that silmitasertib has little effect on the viability of normal colon organoids when used at a concentration that reflects physiological pharmacokinetics observed in clinical studies [21]. In parallel, comparing CKII-specific substrate phosphorylation between mCRC and normal colon PDOs confirmed that CKII signalling activity is higher in the tumour-derived lines (Fig. S5B, C). On the other hand, dinaciclib exerted a marked antitumour effect on both cell and PDO lines; however, a marked reduction in normal colon PDO viability was observed at the same concentration range (Fig. S4D).

Based on these results, we further investigated the molecular differences underpinning the heterogeneous responses to 10 µM silmitasertib (Fig. 3A). As expected [22], western blot quantification revealed a decrease in the phosphorylation levels of CKII substrates (hereafter referred to as “CKII signalling”) following silmitasertib treatment in both groupwise (*p* = 0.001) and paired (*p* = 0.004) comparisons (Fig. 3B, C). Notably, the reduction in CKII signalling strongly correlated with the antitumour activity of silmitasertib (Fig. 3D; *R* = 0.89, *p* = 0.0013), suggesting that the antitumour effect of silmitasertib is related to the suppression of CKII signalling. On the contrary, silmitasertib did not significantly reduce CKII signalling in normal colon PDOs, except for P134, the line with the highest baseline level (Fig. S5D, E). Despite this, it affected neither their viability (Fig. 3A) nor their cell-cycle distribution (Fig. S5F), suggesting that, unlike mCRC PDOs, normal colon epithelium appears less dependent on CKII activity for survival. In parallel, to explore the potential effect of CKII inhibition on primary CRC, we assessed the cytotoxic effect on two PDO lines derived from primary colorectal tumours of an independent cohort (P130 and P255; Table 1). The two lines showed opposite sensitivity to silmitasertib, with P130 being more resistant than P255 (Fig. S4E). Importantly, as observed in mCRC-treated PDOs, the most resistant PDO preserved higher CKII signalling following silmitasertib treatment compared to the more sensitive line (39% ± 9% reduction in P130 vs. 56% ± 4% reduction in P255; Fig. S4F, G). These results highlight the potential therapeutic effect and relevance of silmitasertib across CRC disease stages and the presence of patient-specific resistance to the treatment.

Given the previously described role of CKII in regulating cell cycle progression [23], we examined the impact of silmitasertib on cell cycle regulation. Previous studies have shown that silmitasertib induces G2/M arrest in CRC [24]. We then directly assessed cell cycle status through DAPI-driven flow cytometry analysis. We observed that silmitasertib treatment significantly increased the fraction of cells arrested in the G2/M phase (*p* = 0.008) and, conversely, decreased the fraction of cells in the G1 phase (*p* < 0.05) (Fig. 3E). In particular, silmitasertib-resistant PDOs exhibited a higher increase in the G2/M phase (*R *= −0.67, *p* = 0.046) and a higher reduction in cells in the G1 phase (*R *= −0.75, *p* = 0.020) (Fig. 3F and S4H). On the other hand, cell cycle profiles were not perturbed in the three normal colon PDO lines (Fig. S5F). These observations suggest that the ability of cancer cells to enhance their G2/M checkpoint-related activities, rather than their baseline levels, may be a key determinant of the efficacy of silmitasertib treatment.

Because of CKII involvement in DNA repair and its ability to inhibit caspase-mediated cleavage by phosphorylating protein cleavage sites [25], we hypothesised that silmitasertib treatment may increase both DNA damage and programmed cell death. We therefore assessed the DNA damage status of the PDOs by measuring the levels of the phosphorylated H2AX histone variant (γ-H2AX), a marker for DNA double-strand break (DSB) [26] and the rate of PARP cleavage, a marker of apoptotic cascade activation [27]. The western blot analysis showed that silmitasertib treatment significantly increased DNA damage (*p* = 0.03; Fig. 3G, H) and induced PARP cleavage (*p* = 0.008, Fig. 3G, I), suggesting activation of the apoptotic cascade, with resistant PDOs showing limited PARP cleavage induction after treatment (*R* = 0.65, *p* = 0.060; Fig. 3J). In accordance with CKII’s role in DNA repair mechanisms, we observed a positive correlation between silmitasertib efficacy and DNA repair pathway activity (*R* = 0.78, *p* = 0.013 and *R* = 0.73, *p* = 0.027 for DNA repair and nucleotide excision repair, respectively; Fig. S4I), suggesting that cancer cells with high DNA repair activity might be more sensitive to silmitasertib-mediated CKII inhibition.

Overall, our results demonstrate that silmitasertib exerts heterogeneous antitumour activity in our PDO cohort by interfering with cell cycle dynamics and by promoting apoptosis and DNA damage induction. These data could lay the background for combinatorial treatment between DNA damage-inducing agents and CKII inhibition, a strategy that has been indeed successfully used by Bulanova and colleagues in both ovarian and triple-negative breast cancer [28]. For this reason, we focused our work on different therapeutic strategies, which we explain in the next paragraphs.

### MAPK and Akt-mTOR signalling are linked to silmitasertib’s anticancer efficacy

The previously described observations prompted us to investigate whether parallel mechanisms may limit silmitasertib-mediated CKII pathway suppression.

Pathway enrichment analysis applied to our phosphoproteomic dataset revealed significant enrichment across pathways associated with cell cycle regulation and proliferation in both PDOs and tissues, including the MAPK-related signalling pathways (Fig. S6A). Given the known role of MAPK signalling in promoting cancer development, progression and resistance mechanisms, we decided to further dissect the involvement of this pathway in potentially driving silmitasertib resistance.

**Fig. 4:**
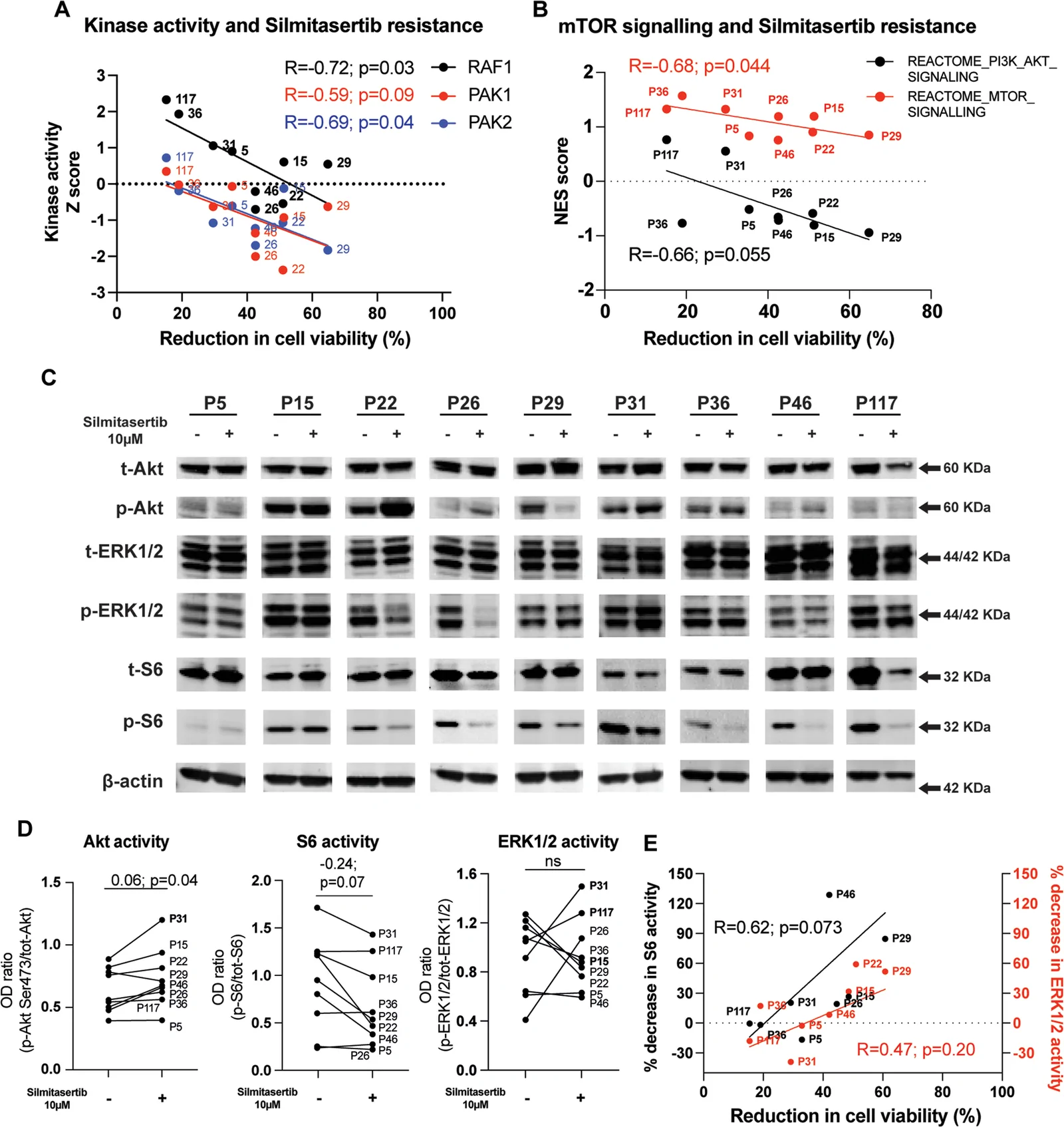
MAPK and Akt-mTOR signalling are linked to silmitasertib antitumour efficacy. See also Fig. S6. (**A**) Scatter plots showing negative correlations between kinase activity Z-scores of RAF1 (black), PAK1 (red) and PAK2 (blue) and reduction in cell viability following silmitasertib treatment across mCRC PDOs (*n* = 9). Pearson’s correlation coefficient (R) and the corresponding p-value are indicated. (**B**) Correlation plot between Reactome gene sets (NES score) and silmitasertib efficacy. NES scores for PI3K-Akt signalling and mTOR signalling are plotted against the reduction in cell viability following silmitasertib treatment across mCRC PDOs (*n* = 9). Pearson’s correlation coefficient (R) and the corresponding p-value are indicated. (**C**) Western blot analysis on the PDO cohort following silmitasertib 10 µM or DMSO. (**D**) Quantification of Akt activity (p-Akt/total Akt), S6 activity (p-S6/total S6), and ERK1/2 activity (p-ERK1/2/total ERK1/2) in PDOs following silmitasertib treatment. Numbers adjacent to significance brackets indicate the median difference. Statistical significance was assessed using the paired Wilcoxon signed-rank test (*n* = 9 PDO lines); *p* = 0.04 for Akt, *p* = 0.07 for S6, ns for ERK1/2. (**E**) Effect of silmitasertib treatment on signalling reduction. The plot shows correlations between silmitasertib antitumour efficacy and the % decrease in S6 (black) and ERK1/2 (red) activity in mCRC PDOs (*n* = 9), calculated relative to the treated condition as (DMSO − silmitasertib)/silmitasertib × 100. The Pearson correlation coefficients are provided: S6 (*R* = 0.62, *p* = 0.073) and ERK1/2 (*R* = 0.47, *p* = 0.20)

We therefore interrogated our (phospho)proteomic dataset to look for correlations between kinase and cancer-related pathway activities and silmitasertib treatment efficacy. We found negative correlations between silmitasertib antitumour efficacy and the activity of RAF1 and PAK1-2 kinases (*R* = −0.72, *p* = 0.03, *R *= −0.59, *p* = 0.09, *R *= −0.69, *p* = 0.04 respectively; Fig. 4A), the latter being direct activators of MEK-driven MAPK and Akt-driven signalling and responsible for CRC progression [29]. Similar observations applied for EGFR and p38 MAPK (known to play a role in CRC progression and therapy resistance [30]) (*R *= −0.65, *p* = 0.06 and *R *= −0.68, *p* = 0.044 respectively; Fig. S6B). Additionally, we also observed that basal PI3K-Akt and mTOR signalling (*R *= −0.66, *p* = 0.055 and *R *= −0.68, *p* = 0.044, respectively, Fig. 4B) negatively correlated with silmitasertib antitumour activity.

These observations prompted us to investigate the influence of MAPK and PI3K-Akt-mTOR signalling on the efficacy of silmitasertib treatment. Using western blot analysis, we assessed the activity of ERK1/2, Akt and S6 protein (Fig. 4C) as proxies for the activity of MAPK and PI3K-Akt-mTOR signalling. Based on the assessment of Akt phosphorylation at Ser473, known to be predictive for Akt-mediated signalling [31], we observed a significant increase in Akt activity following silmitasertib treatment (*p* = 0.04; Fig. 4C, D), suggesting a compensatory activation of upstream Akt signalling. Of note, the PIK3CA-mutated P31 is among the PDOs showing both high basal and increased Akt activity after treatment (Fig. 4D). By contrast, silmitasertib treatment tended to reduce S6 activity, a downstream Akt-mTOR signalling effector (*p* = 0.07, Fig. 4C, D), except in PDOs with low baseline S6 activity (P5 and P26) and those resistant to silmitasertib (P36 and P117). Although other studies have shown that silmitasertib inhibits the PI3K-Akt-mTOR pathway and concomitantly upregulates the MAPK pathway [24, 32], we did not observe a MAPK upregulation post-treatment in our mCRC PDO cohort, as ERK1/2 activity was not affected overall by the treatment (Fig. 4C, D). In particular, we observed high ERK1/2 activity after treatment in KRAS-mutated P31 and P117, but not in the *KRAS-*mutated P15. Conversely, silmitasertib sensitivity showed a positive, although non-significant, correlation with the reduction in S6 activity (*R* = 0.62, *p* = 0.073) and, to a lesser extent, in ERK1/2 activity (*R* = 0.47, *p* = 0.20; Fig. 4E), suggesting that the ability to suppress Akt-mTOR, and to a lesser extent MAPK, signalling may contribute to treatment sensitivity.

Taken together, our data suggest that the ability of mCRC cells to sustain Akt-mTOR and MAPK signalling activity under CKII blockade may represent a potential determinant of silmitasertib resistance.

### Combined MEK inhibition and silmitasertib-mediated CKII blockade exert additive effects on MAPK and Akt-mTOR signalling suppression

**Fig. 5:**
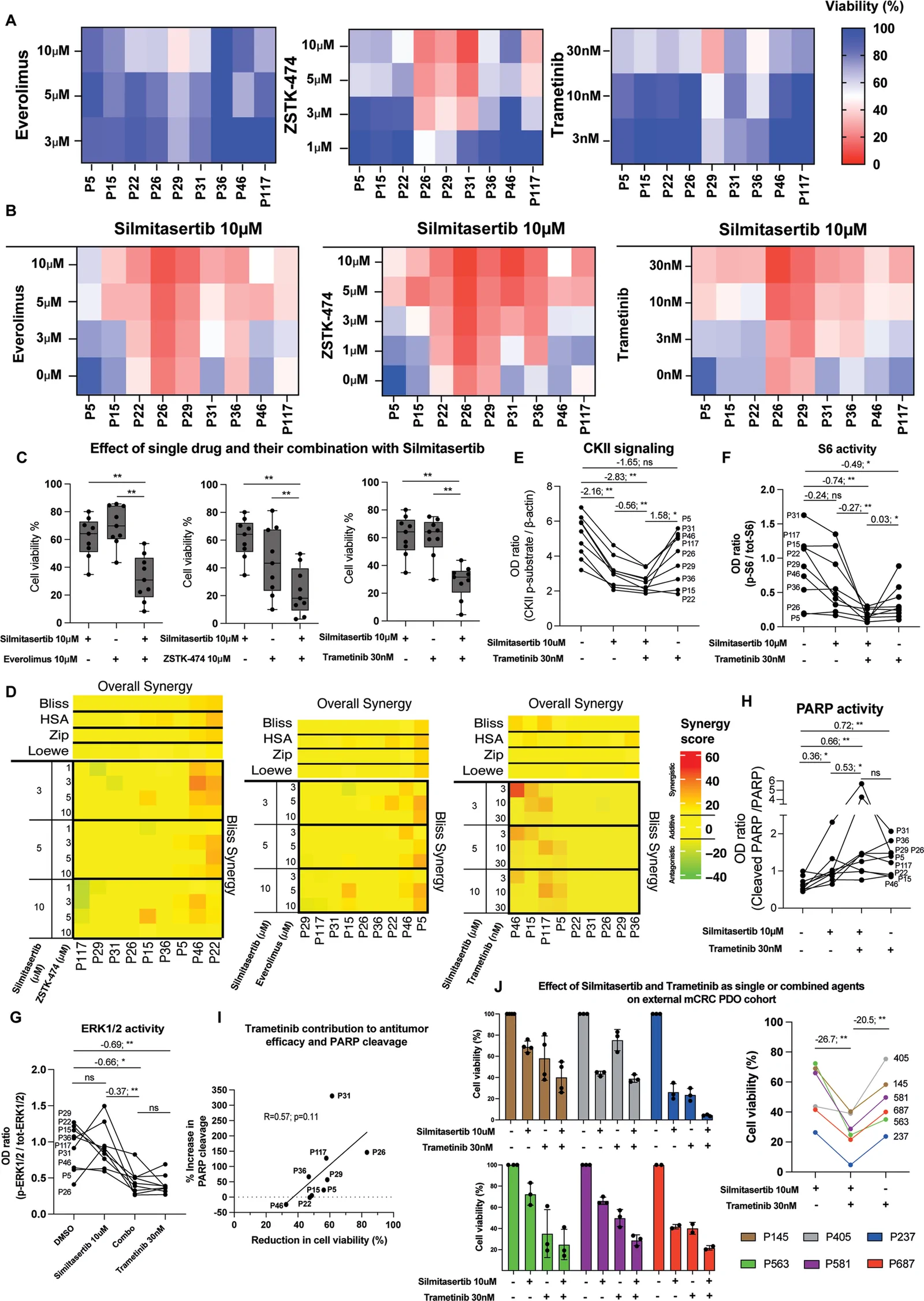
Combined MEK and CKII inhibition enhances anticancer effects in mCRC PDOs. (**A**) Heatmaps showing cell viability (%) of mCRC PDOs treated with increasing concentrations of everolimus (left), ZSTK-474 (middle) and trametinib (right) as single agents. Colour scale represents viability from 0% (red) to 100% (blue). (**B**) Heatmaps displaying cell viability following 10 µM silmitasertib in combination with increasing concentrations of everolimus, ZSTK-474 or trametinib. The bottom row (0 µM/0 nM partner drug) corresponds to silmitasertib 10 µM as a single agent. (**C**) Box plots quantifying cell viability percentages for combinations of 10 µM silmitasertib with everolimus (10 µM), ZSTK-474 (10 µM), or trametinib (30 nM) across the mCRC PDO cohort (*n* = 9 PDO lines). Each dot represents an individual PDO line; boxes indicate the median and interquartile range, and whiskers the minimum and maximum. Statistical significance was assessed using the paired Wilcoxon signed-rank test with Benjamini-Hochberg correction for multiple comparisons (∗∗*p* ≤ 0.01). (**D**) Heatmaps showing the overall synergy between silmitasertib and ZSTK-474 (left), everolimus (middle) or trametinib (right) as measured by the Bliss, HSA, ZIP, and Loewe synergy models. The synergy score displayed in the heatmap matrix is based on the Bliss model. Bliss synergy scores between −10 and +10 indicate additivity; scores >10 indicate synergy and <-10 indicate antagonism. (**E**–**H**) Quantification of CKII signalling (CKII phosphosubstrates/β-actin), S6 and ERK1/2 activity (phosphorylated/total protein) and PARP cleavage (cleaved/total PARP), measured by OD ratio in the mCRC PDO cohort (*n* = 9 PDO lines). See also Fig. S7E, F. Comparisons were performed using the paired Wilcoxon signed-rank test, with p-values adjusted for multiple comparisons using the Benjamini-Hochberg method across the five comparisons shown (ns = not significant, ∗*p* < 0.05, ∗∗*p* < 0.01). Numbers adjacent to significance brackets indicate the median difference. (**I**) Scatter plot showing correlation between trametinib-mediated increase in co-treatment efficacy and PARP cleavage induction across mCRC PDOs (*n* = 9; *R* = 0.57, *p* = 0.11). Pearson’s correlation coefficient (R) and the corresponding p-value are indicated. (**J**) Cell viability of six additional external mCRC PDO lines (P145, P405, P237, P563, P581, P687) treated with DMSO, silmitasertib (10 µM), trametinib (30 nM) or their combination. Left, bars represent mean ± SD of 3 technical replicates from one representative experiment per PDO line. Right, each dot represents the mean viability of one PDO line (*n* = 6); the combination was compared with each single agent by paired two-tailed t-test with Holm correction (∗∗*p* < 0.01). Numbers adjacent to significance brackets indicate the mean difference

Given the link between silmitasertib efficacy and Akt-mTOR and MAPK signalling, we hypothesised that combining silmitasertib-mediated CKII inhibition with the inhibition of either Akt-mTOR or MAPK signalling could enhance antitumour activity in mCRC PDOs. We performed a drug screen combining CKII blockade with either everolimus to inhibit the mTOR pathway; ZSTK-474, a pan-class I PI3K inhibitor with a low affinity for mTOR [33], or trametinib to block MAPK signalling. As for silmitasertib and dinaciclib, we first investigated the dynamic range of drug concentrations on CRC cell lines (Fig. S7A). In the PDOs, the anticancer activity of the co-treatments was significantly stronger than that of their respective single-agent treatments (Fig. 5A-C and S7B), suggesting an additive and/or synergistic interaction between the drugs. In fact, by using SynergyFinder 3.0 [14], we computed an Overall Synergy score using Bliss, HSA, ZIP and Loewe models and found an overall additive interaction for all three tested combinations in most PDOs, with some combinations resulting in a synergistic activity, depending on the applied model (Fig. 5D). The Bliss model is one of the most commonly employed and is recommended for assessing the interaction between two mutually nonexclusive drugs, i.e., when they target different signalling pathways. As shown in Fig. 5D and Additional file 4, most silmitasertib/trametinib concentration combinations were additive (54 of 81), 26 of 81 were synergistic, and a single combination was antagonistic (P26; Bliss score −10.7 at silmitasertib 3 µM and trametinib 10 nM). At the PDO level, the mean Bliss synergy score was additive (−10 < score ≤ 10) in five of nine lines and synergistic in the remaining four (P5, P15, P46 and P117), indicating an interaction ranging from additive to synergistic across PDOs. By contrast, antagonism was more frequent with everolimus (5/81 combinations; 20 synergistic) and ZSTK-474 (9/108; 29 synergistic) (Additional file 4).

We then evaluated the effect on normal colon PDO viability of combining silmitasertib with trametinib or ZSTK-474. Trametinib resulted in no or only a mild reduction in cell viability, both alone and in combination with silmitasertib, while ZSTK-474 resulted in severe reduction of cell viability (Fig. S7C). Everolimus was not pursued further in this context as the concentration we used exceeds clinical dosage [34]. Together with the more frequent antagonism observed with everolimus and ZSTK-474 across individual PDOs, and the superior synergy-to-antagonism ratio achieved with trametinib, these findings supported trametinib as the preferred combination partner. We therefore investigated the molecular consequences of the additive-to-synergistic interaction between silmitasertib and trametinib.

Reflecting what we observed for silmitasertib, we noticed that residual viability following trametinib treatment correlated positively with baseline PI3K-Akt-mTOR signalling (Fig. S7D), indicating that PDOs with high basal pathway activity were less sensitive to the drug. On the other hand, combining silmitasertib with trametinib resulted in a further significant reduction of CKII signalling compared to the individual agents (Benjamini-Hochberg-adjusted *p* = 0.0065 and *p* = 0.015 compared to silmitasertib and trametinib, respectively; Fig. 5E and S7E). Additionally, the combination reduced mTORC1-S6 signalling activity more than either single agent, as indicated by S6 activity assessment (Fig. 5F and S7F), and reduced the expression of RAPTOR, an essential mTORC1 cofactor involved in CRC progression [35], relative to control and to silmitasertib alone (Fig. S7F, G), further highlighting the benefit of the combination. Similar effects were observed for the MAPK pathway, as we observed a significant reduction of ERK1/2 activity following combined treatment (adjusted *p* = 0.033), with trametinib being the driver of this effect (adjusted *p* = 0.0098; Fig. 5G and S7F). Notably, the combination reduced ERK1/2 activity more effectively than silmitasertib alone (adjusted *p* = 0.0098; Fig. 5G). On the other hand, no unidirectional differences were detected on Akt activity after multiple-comparison correction (Fig. S7H). We also noticed that PARP cleavage, i.e., apoptosis induction, was increased by the combination compared with control (adjusted *p* = 0.0098) and with silmitasertib alone (Fig. 5H).

To quantify the trametinib-specific contribution to the combination effect, the percentage change in protein expression, signalling activity and cell viability was calculated by comparing silmitasertib single-agent and co-treatment conditions. Of note, the group of BRAF- and KRAS-mutated PDOs experienced a greater trametinib-mediated decrease in MAPK and PI3K-Akt-mTOR signalling following co-treatment (*p* < 0.05 and *p* = 0.08, respectively; Fig. S7I). The trametinib-mediated increase in co-treatment efficacy also showed a positive, although non-significant, correlation with the corresponding increase in PARP cleavage (*R* = 0.57, *p* = 0.11; Fig. 5I). To substantiate these findings, apoptosis induction was independently validated by quantifying caspase-3/7 activity in a representative subset of PDOs, comprising four lines selected among the most sensitive (P26, P29) and the most resistant (P31, P117) models. As shown in Fig. S7J, co-treatment with both drugs significantly elevated caspase-3/7 activity levels. In agreement with the PARP cleavage data (Fig. 5H and S7F), the most pronounced apoptotic response was observed in the P26 line. In contrast, no significant changes in caspase-3/7 activity were observed in normal colon PDOs under any treatment condition (Fig. S5G).

Finally, to further demonstrate the anticancer efficacy of the proposed drug combination in mCRC PDOs, we extended our analysis to six additional PDO lines established from the liver metastases of an independent cohort of mCRC patients (Table 1). As illustrated in Fig. 5J, co-treatment with silmitasertib and trametinib reduced cell viability in all six external mCRC PDO lines. Across the six lines, the combination reduced viability more than silmitasertib (mean difference −26.7%, adjusted *p* = 0.007) and trametinib (−20.5%, adjusted *p* = 0.004) alone. The added benefit varied between lines (Fig. 5J, left); this heterogeneous pattern is consistent with the predominantly additive interaction between the two drugs observed across the primary PDO cohort.

Taken together, these data indicate that trametinib-driven suppression of MAPK and Akt-mTOR signalling cooperates with silmitasertib-mediated CKII blockade to enhance anticancer efficacy in mCRC PDOs.

### Silmitasertib and trametinib co-treatment reduces the active β-catenin abundance and the CRC stem cell pool

**Fig. 6:**
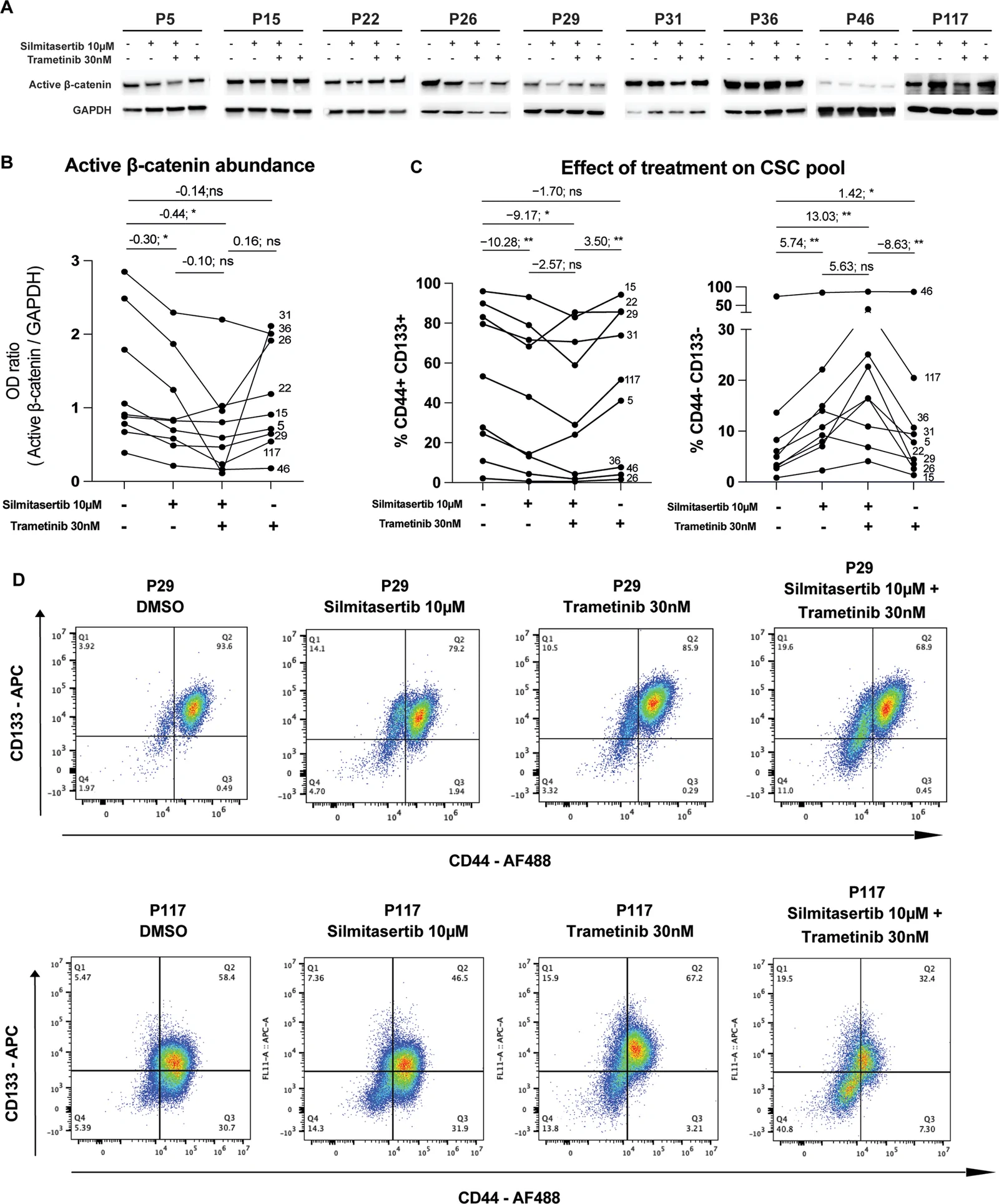
Silmitasertib and trametinib co-treatment reduces active β-catenin abundance and decreases the CRC stem cell pool. (**A**) Western blot analysis of active β-catenin (non-phosphorylated, Ser33/37/Thr41). (**B**) Paired plot quantifying active β-catenin abundance (OD ratio to GAPDH) across four treatment conditions (DMSO, silmitasertib, trametinib, and their combination) in nine mCRC PDO lines. Comparisons were performed using the paired Wilcoxon signed-rank test, with p-values adjusted for multiple comparisons using the Benjamini-Hochberg method across the five comparisons shown (ns = not significant, *p < 0.05, **p < 0.01). Numbers adjacent to significance brackets indicate the median difference. (**C**) Analysis of cancer stem cell population abundance, measured as the percentage of CD44+ CD133+ or CD44− CD133− cells across four treatment conditions (DMSO, silmitasertib, trametinib, and their combination) in nine mCRC PDO lines, as assessed by flow cytometry. Comparisons were performed using the paired Wilcoxon signed-rank test, with p-values adjusted for multiple comparisons using the Benjamini-Hochberg method across the five comparisons shown (ns = not significant, *p < 0.05, **p < 0.01). Numbers adjacent to significance brackets indicate the median difference. (**D**) Representative flow cytometry plots showing the effect of respective treatment conditions on CD44 and CD133 marker expression in P29 (top row) and P117 (bottom row). Percentages indicate the fraction of cells in each quadrant

Considering the established role of β-catenin/Wnt signalling in CRC maintenance and progression [36] and the regulatory role that CKII exerts on this pathway, we investigated the impact of our co-treatment on this signalling cascade. Interestingly, the strongest combination effect between the two drugs was observed in PDOs exhibiting the highest basal Wnt activity (*R* = 0.74, *p* = 0.021; Fig. S8A). Given that β-catenin is a core component of the cadherin protein complex and that its stabilisation is essential for Wnt signalling activation, we assessed the abundance of the active β-catenin isoform via western blot (Fig. 6A). The analysis revealed a significant reduction in active β-catenin levels following silmitasertib treatment and its combination with trametinib (median −0.30 and −0.44; Benjamini-Hochberg-adjusted *p* = 0.020 and *p* = 0.029, respectively; Fig. 6B), whereas trametinib alone had no significant effect. β-catenin tended to be less active under the combination than under either single agent (median difference −0.10 versus silmitasertib and −0.16 versus trametinib); however, these differences did not reach statistical significance after correction for multiple comparisons (both adjusted *p* = 0.068), consistent with an additive rather than synergistic interaction between the two drugs. Co-expression of CD44 and CD133 in CRC cells has been proposed to mark the pool of CRC stem cells (CSCs), with the Wnt/β-catenin pathway being over-activated in this population [37, 38]. In fact, the pharmacological inhibition of the Wnt pathway decreases both CD133 [39] and CD44 expression and suppresses the tumour-initiating properties of CD44+ CD133+ CRC cells [38]. Moreover, CD44 was shown to be essential for canonical Wnt signalling activation [40]. Therefore, we investigated the expression of these markers to monitor the effects of treatments on CSCs. Our results showed that the co-treatment significantly reduced the pool of CD44+ cells compared with control and with either single agent (Fig. S8B), further suggesting a reduced Wnt/β-catenin pathway activity following combined treatment. When examining the effects on the double-positive population, we observed that both silmitasertib and its combination with trametinib significantly reduced the CD44+ CD133+ cancer stem cell pool (median −10.28 and −9.17; Benjamini-Hochberg-adjusted *p* = 0.0098 and *p* = 0.033, respectively), whereas trametinib alone had no significant effect. Concomitantly, the CD44− CD133− double-negative population increased significantly under silmitasertib and the combination (median +5.74 and +13.03; adjusted *p* = 0.0065 for both) and, to a lesser extent, under trametinib alone (median +1.42; adjusted *p* = 0.015), indicating a shift away from the stem-like compartment (Fig. 6C and Additional file 5). However, as expected, a heterogeneous outcome was observed in our cohort. Examples of CD44 and CD133 expression as measured via flow cytometry are shown in Fig. 6D. Finally, we performed qPCR analysis of Wnt target genes and stem cell markers LGR5, OLFM4 and AXIN2 in a subset of PDOs. Our results (Fig. S8C) show that the silmitasertib/trametinib combination reduced or prevented upregulation of AXIN2, LGR5 and OLFM4 in most tested PDOs. These findings align with the reduced abundance of the active β-catenin isoform following combined treatment and collectively support the conclusion that combined CKII and MEK inhibition attenuates Wnt pathway activity and reduces the CRC stem cell pool. This is important to highlight, as other work showed an increased Wnt signalling and stem cell activity following anticancer therapy, which drives therapy resistance [41].

Overall, these findings suggest that the co-treatment attenuates Wnt signalling and reduces the CSC compartment in mCRC PDOs.

## Discussion

Although the prognosis for mCRC patients has significantly improved over the past decades [42], drug resistance remains a major clinical challenge, underscoring the need for novel therapeutic strategies. PDOs have become an optimal platform for drug discovery, and proteogenomic studies have expanded our ability to dissect cancer vulnerabilities and to design ad hoc therapeutic strategies [43]. In this study, we aimed to identify new personalised medicine approaches for mCRC patients through the analysis of patients’ perturbed phosphoproteome networks and the use of mCRC PDOs.

Our primary finding is the consistent upregulation of casein kinase II activity in both CRC tissues and PDOs. CKII is a constitutively active serine/threonine protein kinase involved in regulating pathways such as DNA damage response and cell cycle progression [23, 25]. Through a mechanism known as “non-oncogene addiction” [44], CKII drives cancer progression and metastasis and contributes to drug resistance via the control of drug efflux, DNA repair and key signalling pathways such as NF-κB, β-catenin and p53 [24].

The growing evidence concerning the role of CKII in cancer has intensified efforts to develop specific inhibitors [45]. Silmitasertib is an ATP-competitive inhibitor of CKII catalytic subunits that received FDA orphan drug designation, first for cholangiocarcinoma. While the development of more selective CKII inhibitors, such as SGC-CK2-1 [46], has raised questions about the anticancer specificity of silmitasertib, two independent phosphoproteomic investigations in both healthy and cancer cell lines demonstrated that most of the biological processes perturbed by silmitasertib are attributable to direct CKII inhibition rather than off-target effects [47, 48].

We found that silmitasertib treatment increased G2/M arrest across our PDO cohort, consistent with previously reported effects [24]. Notably, this effect was more pronounced in resistant PDOs, suggesting that enhanced G2/M checkpoint engagement may represent a survival mechanism in resistant cells rather than a cytotoxic response. These observations could pave the way for new therapeutic strategies targeting G2/M-related pathways to enhance sensitivity to silmitasertib inhibition [49]. Furthermore, given the role of CKII in phosphorylating key constituents of DNA damage and repair pathways [50], our correlative data suggest that cancer cells with high baseline DNA repair activity may be more susceptible to silmitasertib-mediated CKII inhibition, though this hypothesis warrants further experimental validation.

PI3K-Akt-mTOR signalling is required for the malignant behaviour of CKII-overexpressing gastric cells [51]. Silmitasertib has been shown to inhibit the PI3K-mTOR-S6K while simultaneously inducing MAPK signalling through various post-translational and epigenetic mechanisms, collectively leading to the activation of apoptosis or methuosis [24, 32]. While our data showed a trend towards reduced S6 activity, consistent with suppression of the PI3K-mTOR-S6K pathway, we did not observe a corresponding increase in MAPK activity. However, we observed that ERK1/2 activity was maintained in resistant PDOs following silmitasertib treatment, suggesting that persistent MAPK signalling may contribute to resistance. Interestingly, we observed that the maintenance of ERK1/2 activity following silmitasertib treatment was particularly evident in KRAS-mutated PDOs derived from patients who had received prior neoadjuvant chemotherapy (P31, P117), but not in the treatment-naive P15, suggesting that treatment history may influence the rewiring of MAPK signalling. However, given the limited sample size, this observation warrants further investigation.

Overall, by integrating phosphoproteomic data with molecular investigation and drug treatment outcomes, our work suggests that silmitasertib efficacy is associated with the modulation of MAPK and Akt-mTOR-S6 signalling. Concordantly, combining silmitasertib with MAPK (e.g. trametinib) or PI3K-Akt-mTOR (e.g. ZSTK-474) inhibition resulted in predominantly additive drug interactions, synergistic in some PDOs, that significantly enhanced anticancer efficacy. Importantly, we found that the silmitasertib/trametinib combination, which had minimal impact on normal colon PDO viability, simultaneously reduces both the MAPK and Akt-mTOR-S6 signalling pathways. We also found that high baseline PI3K-Akt-mTOR signalling correlates with resistance to both silmitasertib and trametinib when used as single agents, providing a rationale for their combination. While the effect of combining CKII and MEK inhibition has been previously described [52, 53], we reported here for the first time that trametinib cooperates with silmitasertib in further reducing CKII activity. An intriguing aspect that warrants further investigation is how trametinib exerts this secondary effect on Akt-mTOR signalling reduction. Evidence points to a role for RSK-mediated phosphorylation of Raptor to mediate a MAPK-dependent mTORC1 activation [54], suggesting that trametinib may exert its effect by disrupting this critical linkage pathway.

Although synergy is often the preferred readout in drug-combination studies, it is neither a necessary nor a common property of clinically effective combinations: indeed, 95% of FDA-approved combinations for advanced cancers show additive or less-than-additive effects on progression-free survival [55]. The predominantly additive, and in some PDOs synergistic, interaction we observed between silmitasertib and trametinib therefore supports further preclinical evaluation of this combination. However, as the interaction type varied with concentration in some PDOs, small differences in drug exposure could shift it towards synergy or antagonism, warranting dose-optimisation studies before clinical translation.

*APC* is frequently mutated in CRC and is responsible for driving Wnt signalling activation through the aberrant stabilisation of β-catenin, which is essential for stem cell self-renewal in both healthy adult stem cells and CSCs [56]. CD44 and CD133 proteins have been demonstrated to confer important tumour-initiating features in CSC and to drive resistance to cancer therapy [37, 38]. Our finding that the combination of silmitasertib and trametinib effectively reduces active β-catenin abundance and decreases the CRC stem cell pool is relevant because trametinib alone has been shown to inadvertently promote cell plasticity via Wnt signalling activation [41], further emphasising the importance of combination strategies.

Our study has limitations. While the cohort of ten mCRC PDOs, nine of which were available for functional experiments, was extensively characterised by matched multi-omic profiling and complemented by a validation panel of six additional PDOs, confirmation in larger independent cohorts is warranted. A further limitation is that the normal colon mucosa tissues used as reference for the (phospho)proteomic comparisons were obtained from unrelated patients rather than from tissue from the same tumour donors, as tumour specimens were surgically resected from liver metastases and matched normal colon mucosa was not accessible. Consequently, part of the observed tumour-versus-normal differences may reflect inter-patient heterogeneity rather than strictly tumour-specific alterations. Patient-derived organoids lack the immune and stromal components of the tumour microenvironment, which may modulate the response to CKII and MEK inhibition and should be evaluated in immune-competent and co-culture systems. Finally, the therapeutic potential of the silmitasertib/trametinib combination requires validation in preclinical in vivo models before clinical translation can be considered.

## Conclusions

In summary, our phosphoproteomic-guided approach identified CKII as a therapeutic vulnerability in mCRC and provided a rationale for combining silmitasertib with trametinib. Further validation in preclinical in vivo models and clinical settings will be essential to establish the full therapeutic potential of this combination for mCRC patients.

**Table 1:** Clinical and molecular characteristics of patient-derived tumour and normal colon samples

	Patient no.	Age	Sex	Histological characterisation	Site of primary tumour	Pre-treatment	MSI/MSS status	KRAS status	BRAF status
mCRC patients 1st cohort	5	69	M	Adenocarcinoma	Colon Sigmoideum	XELOX	MSS	wt	wt
	15	81	F		Colon caecum	No treatment	MSS	G12D	wt
	17	61	F		Colon Sigmoideum	FOLFOX + Cetuximab	MSS	wt	wt
	22	79	F		Colon Ascendens	FOLFOX	MSI	wt	V600E
	26	62	F		Colon Descendens / Sigmoideum	XELOX	MSS	wt	wt
	29	60	M		Colon Sigmoideum	FOLFOX + Cetuximab	MSS	wt	wt
	31	60	M		Colon Ascendens	FOLFOX/FOLFIRI	MSS	G12D	wt
	36	57	M		Rectum	FOLFIRI/Panitumumab	MSS	wt	wt
	46	66	M		Colon caecum	FOLFOX + Cetuximab	MSS	wt	wt
	117	50	F		Colon Sigmoideum	FOLFOX/FOLFIRI	MSS	G13D	wt
mCRC patients 2nd cohort	145	78	M	Adenocarcinoma	Colon transversum	FOLFOX	MSS	wt	wt
	237	72	M		Colon caecum	FOLFOX	MSS	G12D	wt
	405	82	M		Colon (left)	–	–	wt	wt
	563	64	F		Colon Sigmoideum	FOLFOX + panitumumab, then 5-FU/folinic acid + panitumumab	MSS	wt	wt
	581	71	M		Colon Sigmoideum	XELOX + Bevacizumab	MSS	Q61H	wt
	687	77	M		Rectum	XELOX + Bevacizumab	MSS	G12D	wt
Primary CRC PDOs	130	77	M	Adenocarcinoma	Colon caecum	No treatment	MSS	wt	wt
	255	78	F		Colon Sigmoideum	No treatment	MSS	wt	wt
Normal colon PDOs	134	62	F	Normal Mucosa	–	No treatment	–	–	–
	135	73	F	Normal Mucosa	–	No treatment	–	–	–
	645	51	M	Normal Mucosa	–	No treatment	–	–	–
Normal colon tissues	323	61	M	Normal Mucosa	–	No treatment	–	–	–
	327	66	M		–	No treatment	–	–	–
	329	78	F		–	No treatment	–	–	–
	336	84	F		–	No treatment	–	–	–
	337	81	F		–	No treatment	–	–	–
	338	76	F		–	No treatment	–	–	–

First cohort: PDOs and matched tissues derived from mCRC liver metastases, used for (phospho)proteomic profiling and drug screening; second cohort: PDOs derived from mCRC liver metastases, used for validation (Fig. 5J); primary CRC PDOs: PDO lines derived from primary colorectal tumours (Fig. S4E–G); normal colon PDOs: PDO lines established from normal colon mucosa; normal colon tissues: normal colon mucosa used as (phospho)proteomic reference. For both metastatic cohorts, the site of the primary tumour is reported for clinical annotation only; all tissues and organoids were obtained from the resected liver metastasis. Abbreviations: M=male; F=female; XELOX=capecitabine + oxaliplatin; FOLFOX=folinic acid + fluorouracil + oxaliplatin; FOLFIRI=folinic acid + fluorouracil + irinotecan; 5-FU=fluorouracil; cetuximab and panitumumab=anti-EGFR monoclonal antibodies; bevacizumab=anti-VEGF monoclonal antibody; MSS/MSI=microsatellite stable/unstable; wt=wild-type; -=not available

## Supplementary Information

Below is the link to the electronic supplementary material.

Additional file 1. Supplementary Methods (including Supplementary References).

Additional file 2. Supplementary Table 1 (IHC, western blot, Flow cytometry, Drugs and Primers).

Additional file 3. Supplementary Figures (S1-S8).

Additional file 4. Supplementary Table 2 (Synergy scores).

Additional file 5. Supplementary Table 3 (CD44 and CD133 values).

## Data Availability

Processed proteomic, phosphoproteomic and whole-exome sequencing data have been deposited in Zenodo (DOI 10.5281/zenodo.22960021).

## Abbreviations

mCRC: Metastatic colorectal cancer
CRC: Colorectal cancer
PDO: Patient-derived organoid
CKII: Casein kinase II
KSEA: Kinase-substrate enrichment analysis
GSEA: Gene set enrichment analysis
WES: Whole-exome sequencing
MSS/MSI: Microsatellite stable/unstable
SD: Standard deviation
APC: Adenomatous polyposis coli
CSC: Cancer stem cell
AKT: Protein Kinase B
PI3K: Phosphatidylinositol 3-kinase
mTOR: Mammalian Target Of Rapamycin
mTORC1: mTOR Complex 1
MAPK: Mitogen-Activated Protein Kinase
ERK1/2: Extracellular Signal-Regulated Kinase 1/2
S6: Ribosomal Protein S6
CD44: Cluster of Differentiation 44
CD133: Cluster of Differentiation 133
LGR5: Leucine-rich repeat-containing G-protein-coupled receptor 5
OLFM4: Olfactomedin 4
AXIN2: Axis Inhibition Protein 2
MEK: Mitogen-Activated Protein Kinase Kinase
Wnt: Wingless-related Integration site
PARP: Poly(ADP-Ribose) Polymerase
FDA: Food and Drug Administration
NF-κB: Nuclear Factor kappa-light-chain-enhancer of activated B cells
KRAS: Kirsten Rat Sarcoma viral proto-oncogene
BRAF: B-Raf proto-oncogene, serine/threonine kinase
RAPTOR: Regulatory-Associated Protein of mTOR
NELFE: Negative Elongation Factor E
H&E: Haematoxylin and eosin
IHC: Immunohistochemistry
HSA: Highest single agent
ZIP: Zero interaction potency
MSigDB: Molecular Signatures Database
OD: Optical density
PFA: Paraformaldehyde
RT-qPCR: Reverse transcription quantitative polymerase chain reaction
NCM460: Normal Colonic Mucosa 460
TP53: Tumour Protein p53
MYC: Myelocytomatosis viral oncogene homolog
CDX2: Caudal-type homeobox transcription factor 2
CK20: Cytokeratin 20
MLH1: MutL Homolog 1
PMS2: PMS1 Homolog 2
qPCR: Quantitative Polymerase Chain Reaction
γ-H2AX: γ-Histone 2 A variant X
DSB: Double-Strand Break
PAK1-2: p21-Activated Kinase 1 and 2
EGFR: Epidermal Growth Factor Receptor
VEGF: Vascular Endothelial Growth Factor
FOLFOX: Folinic acid + fluorouracil + oxaliplatin
XELOX: Capecitabine + oxaliplatin
FOLFIRI: Folinic acid + fluorouracil + irinotecan
DAPI: 4’,6-Diamidino-2-phenylindole
PIK3CA: Phosphatidylinositol 3-kinase catalytic subunit alpha
DMSO: Dimethyl sulfoxide
NES: Normalised enrichment score
LC-MS/MS: Liquid chromatography-tandem mass spectrometry
pS/pT: Phosphoserine/phosphothreonine
