# Supplementary material for: Combined casein kinase II and MAPK signalling inhibition as a therapeutic strategy for metastatic colorectal cancer
Source: Exp Hematol Oncol. 2026 Nov 5;15:119. doi: 10.1186/s40164-026-00855-4 (PMC13737822; doi:10.1186/s40164-026-00855-4)
Supplement: Supplementary file 1 [file 40164_2026_855_MOESM3_ESM.pdf]

Additional File 3: Supplementary Figures

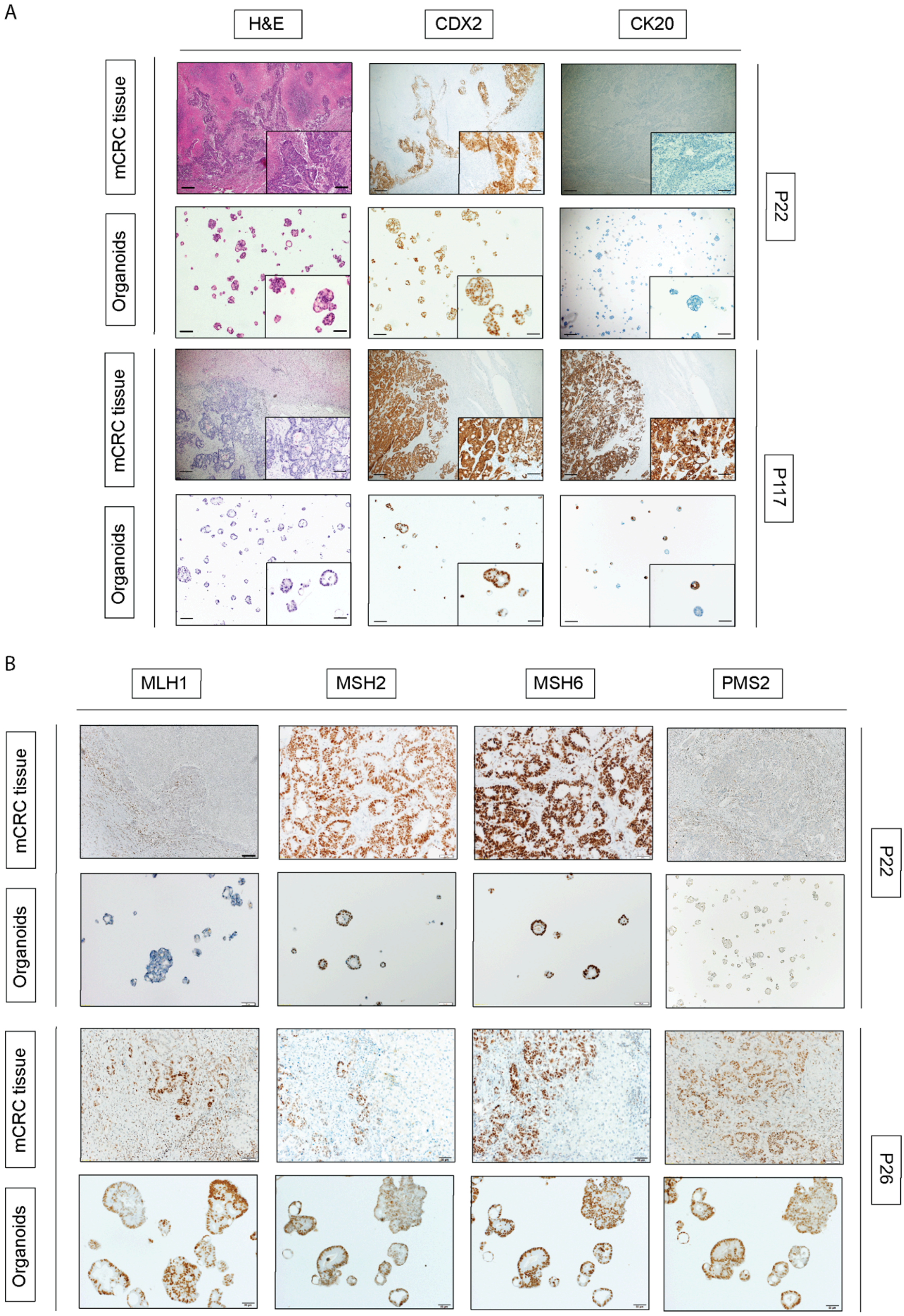

Figure S1. Establishment and Characterisation of Patient-Derived Organoids from Metastatic Colorectal Cancer tissues. (**A**) Representative images of H&E and IHC staining for CDX2 and CK20 in two mCRC tumour tissues and matched PDOs, complementing Figure 1A. (**B**) Representative IHC staining of mismatch repair proteins, MLH1, MSH2, MSH6, and PMS2, in two mCRC tumour tissues and matched PDOs. Both P22 tissue and PDO lack MLH1 and PMS2 protein expression, indicating an MSI-high status. Insets highlight regions of interest. For tissue pictures, scale bars indicate 100 μm and 25 μm for lower and higher magnifications, respectively. For PDO pictures, scale bars are 50 μm and 10 μm for lower and higher magnifications, respectively.

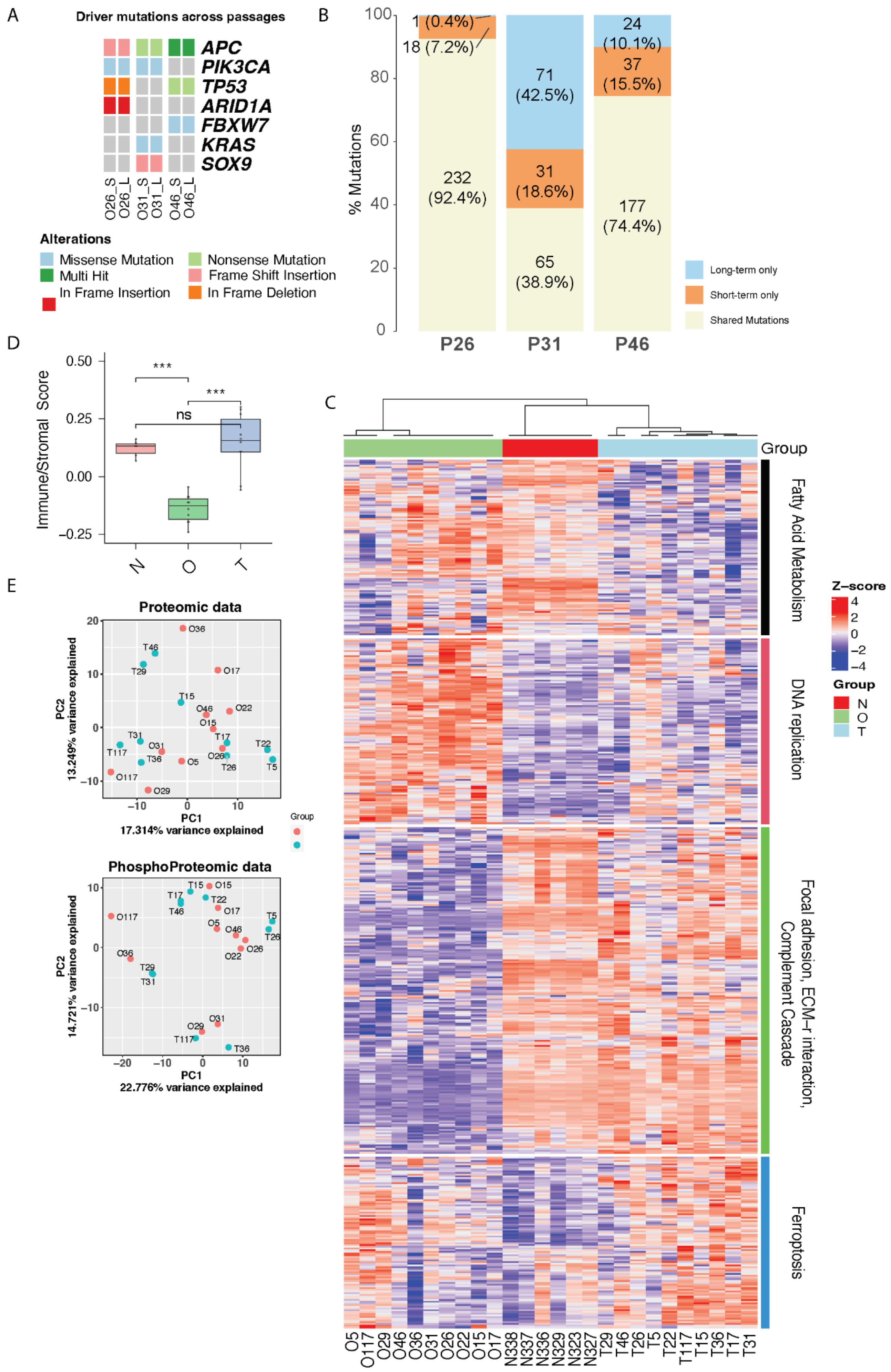

A
Driver mutations across passages
APC
PIK3CA
TP53
ARID1A
FBXW7
KRAS
SOX9
O26_S O26_L O31_S O31_L O46_S O46_L
Alterations
Missense Mutation
Nonsense Mutation
Multi Hit
Frame Shift Insertion
In Frame Insertion
In Frame Deletion
B
% Mutations
1 (0.4%)
18 (7.2%)
232 (92.4%)
71 (42.5%)
31 (18.6%)
65 (38.9%)
24 (10.1%)
37 (15.5%)
177 (74.4%)
P26
P31
P46
Long-term only
Short-term only
Shared Mutations
C
Group
Fatty Acid Metabolism
DNA replication
Focal adhesion, ECM–r interaction, Complement Cascade
Ferroptosis
Z–score
Group
N
O
T
O5 O117 O29 O46 O36 O31 O26 O22 O15 O17 N338 N337 N336 N329 N323 N327 T29 T46 T26 T5 T22 T117 T15 T36 T17 T31
D
Immune/Stromal Score
***
***
ns
N
O
T
E
Proteomic data
PC2
13.249% variance explained
PC1
17.314% variance explained
Group
PhosphoProteomic data
PC2
14.721% variance explained
PC1
22.776% variance explained

Figure S2. Driver mutation stability and (phospho)proteomic comparison of mCRC PDOs and matched tissues. (**A**) Oncoplot showing mutational profiles of key colorectal cancer driver genes across multiple organoid passages (S=Short-term; L=Long-term). Colour bars represent the types of mutations, as indicated in the legend. (**B**) Stacked bar plots showing the concordance of all somatic variants detected in short- and long-term cultures of P26, P31 and P46. For each PDO, variants called in either passage were genotyped in both samples using GATK and considered present when supported by at least two alternative reads. Bars indicate the percentage of variants shared between passages (beige) or detected only in the short-term (orange) or long-term (blue) culture; numbers within bars indicate absolute variant counts and percentages. (**C**) Consensus hierarchical clustering of the 500 most variable proteins, showing Z-scores of protein abundance across mCRC tumours (T), PDOs (O) and normal colon tissues (N). Pathways enriched by KEGG analysis are annotated per k-means cluster of proteins. (**D**) Box plot comparing the overall immune/stromal score derived from ConsensusTME deconvolution on the proteomics data among normal colon tissue (N), tumour organoids (O), and mCRC tumours (T) (N, n=6; O, n=10; T, n=10). Each dot represents one sample; boxes indicate the median and interquartile range, and whiskers extend to 1.5× the interquartile range. Statistical significance was determined using Mann–Whitney U tests (***p<0.001, ns=not significant). (**E**) Principal component analysis (PCA) plots depicting the variance explained by principal components (PC1, PC2) of the 500 most variable features for proteomic and phosphoproteomic data across mCRC tumours (T) and PDOs (O). Each dot represents a sample, colour-coded by group.

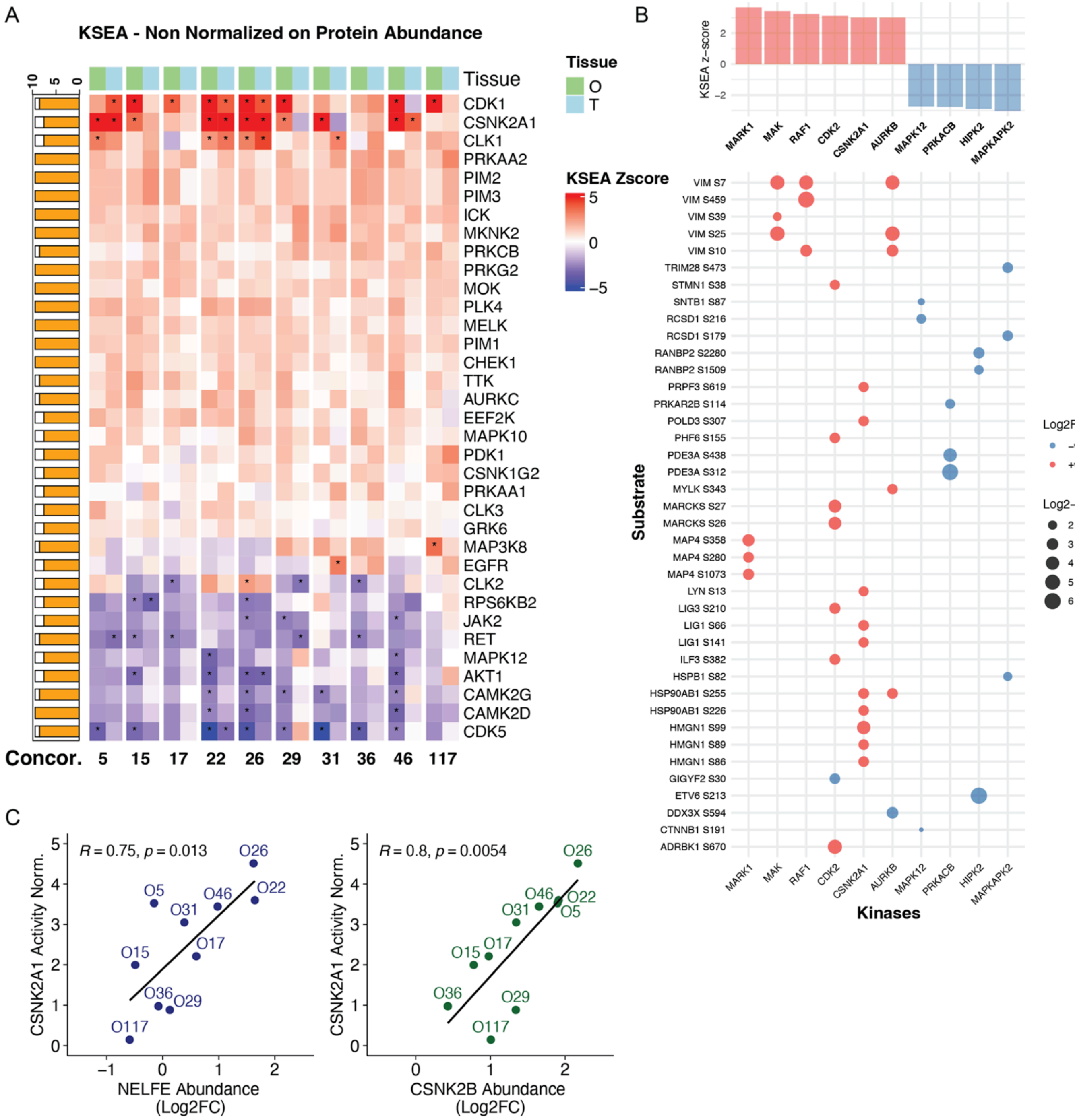

Figure S3. CKII activity is upregulated in metastatic CRC tissues and PDOs. (**A**) Heatmap showing KSEA based on phosphosubstrate levels without normalisation on protein abundance in PDOs and mCRC tumour samples. The colour scale represents the KSEA Z-score, indicating kinase activity across different samples (green=organoids, blue=tumours). Enrichment concordance (Concor., orange bars on the left) indicates, for each kinase, the number of matched tumour/PDO pairs showing the same direction of enrichment (scale 0-10). (**B**) Bubble plot in the lower panel showing the top differentially phosphorylated substrates in PDOs (y-axis) compared to the normal colon pool reference. The size of the bubbles represents the fold change (Log_2FC) in phosphosubstrate abundance, with positive and negative fold changes indicated by red and blue bubbles, respectively. These parameters are used to compute the KSEA Z-score for each kinase, displayed as bars in the upper part of the panel. (**C**) Scatter plots showing correlations between CSNK2A1 (CKII alpha) activity and NELFE (left) and CSNK2B (CKII beta) protein abundance (right) in PDOs compared to normal colon tissue (n=10). The Pearson correlation coefficient (R) and p-values are indicated, showing significant positive correlations (R=0.75, p=0.013 for NELFE; R=0.8, p=0.0054 for CSNK2B).

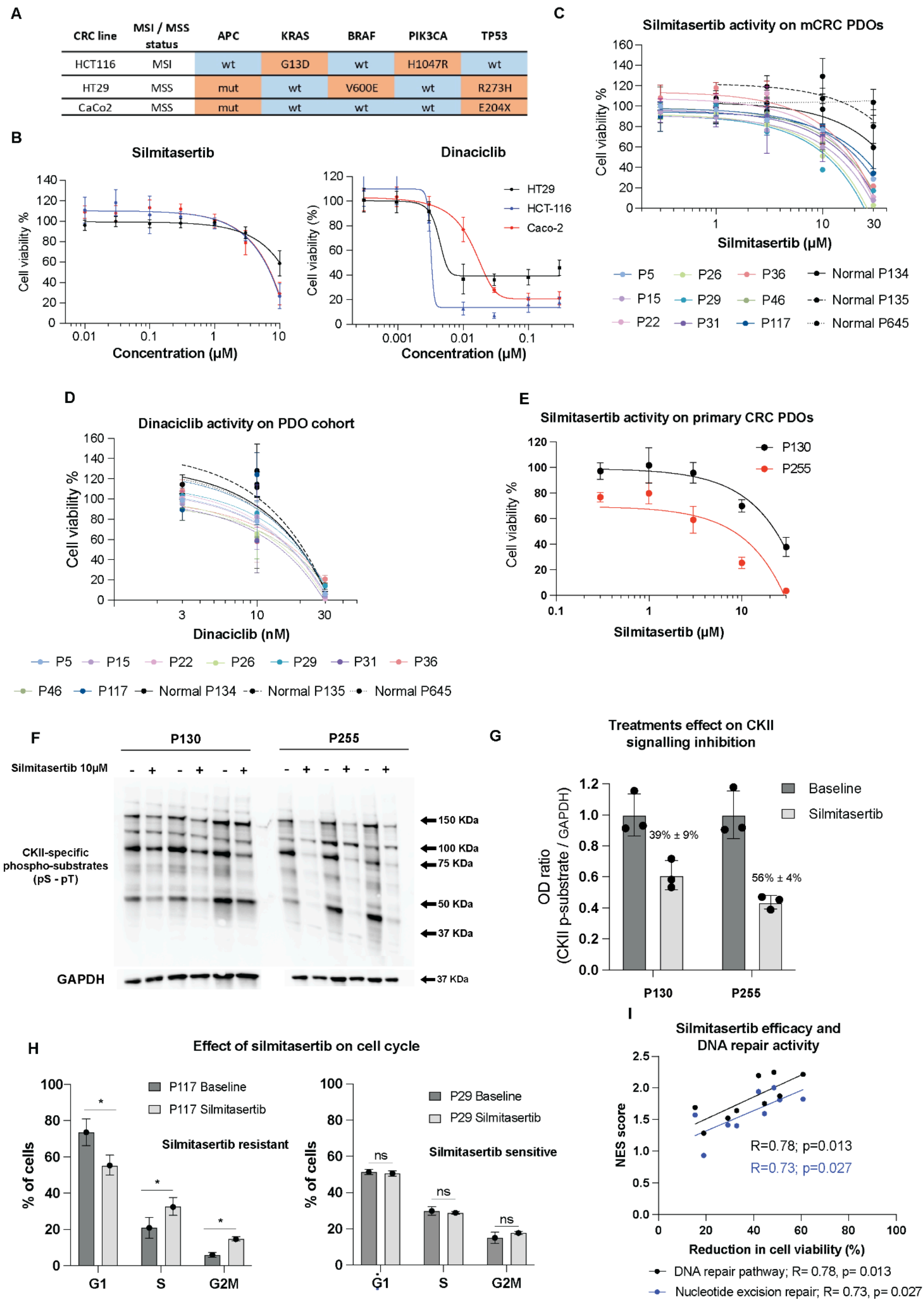

CRC line	MSI / MSS status	APC	KRAS	BRAF	PIK3CA	TP53
HCT116	MSI	wt	G13D	wt	H1047R	wt
HT29	MSS	mut	wt	V600E	wt	R273H
CaCo2	MSS	mut	wt	wt	wt	E204X

Figure S4. Silmitasertib interferes with cell cycle activity and exerts antitumour effects in primary and mCRC PDOs. (**A**) Summary of the driver mutation characteristics of HCT116, HT-29 and CaCo2 CRC cell lines, including MSI/MSS status. (**B**) Dose-response curves showing the effect of silmitasertib (left) and dinaciclib (right) on cell viability on CRC cell lines. (**C**) Dose-response curves showing the effect of silmitasertib and (**D**) dinaciclib on cell viability across the PDO cohort (n=9) together with normal colon PDOs (n=3). The panels highlight the lower sensitivity of normal colon PDOs to silmitasertib, especially at a 30 μM concentration and, conversely, marked sensitivity to dinaciclib. (**E**) Dose-response curves of silmitasertib in two primary CRC PDOs. In (**B–E**), data are presented as mean ± SD of at least three independent experiments performed in triplicate; curves were fitted by nonlinear regression. (**F**) Western blot and its quantification (**G**) showing CKII-specific phosphosubstrate levels (pSer/pThr motif) and GAPDH loading control in primary CRC PDOs treated with silmitasertib 10 μM or DMSO. In (**G**), bars represent the mean ± SD of three independent biological replicates per line, each dot represents one replicate, and the mean percentage reduction ± SD is indicated. (**H**) Bar plots showing percentage of cells in G1, S, and G2/M phases in silmitasertib-resistant P117 (left) and silmitasertib-sensitive P29 (right) before and after silmitasertib treatment. Bars represent the mean ± SD of three independent experiments. Statistical significance was determined by an unpaired two-tailed t-test (*$p<0.05$; ns=not significant). (**I**) Scatter plots showing positive correlations between silmitasertib antitumour efficacy and baseline DNA repair pathway (black, R=0.78, p=0.013) and nucleotide excision repair (blue, R=0.73, p=0.027) activity scores across mCRC PDOs (n=9). Pearson's correlation coefficient (R) and the corresponding p-value are indicated.

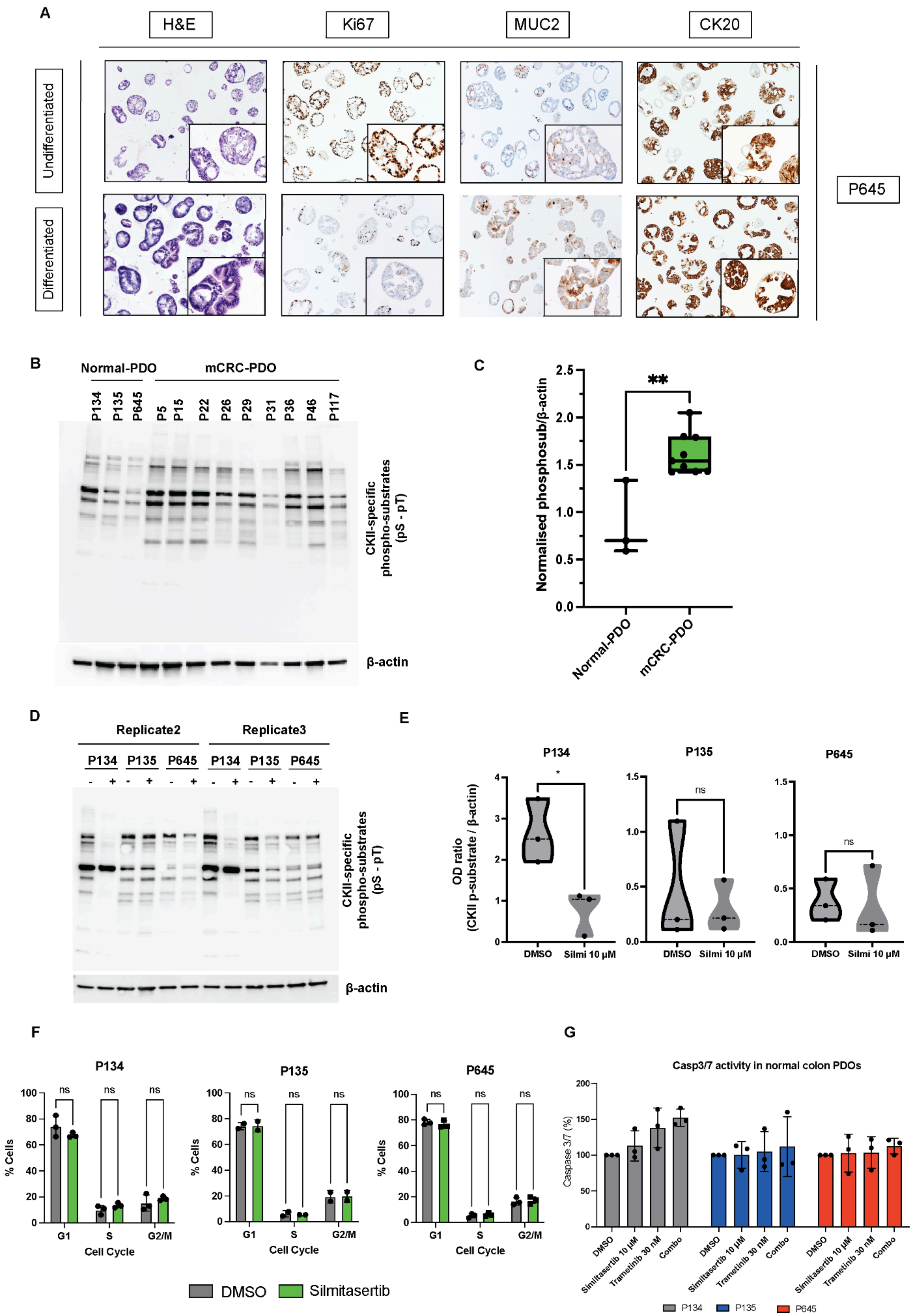

A
H&E
Ki67
MUC2
CK20
Undifferentiated
Differentiated
P645
B
Normal-PDO
mCRC-PDO
P134 P135 P645 P5 P15 P22 P26 P29 P31 P36 P46 P117
CKII-specific phospho-substrates (pS - pT)
β-actin
C
Normalised phosphosub/β-actin
**
Normal-PDO
mCRC-PDO
D
Replicate2
Replicate3
P134 P135 P645 P134 P135 P645
CKII-specific phospho-substrates (pS - pT)
β-actin
E
P134
P135
P645
OD ratio (CKII p-substrate / β-actin)
*
ns
ns
DMSO
Silmi 10 μM
F
P134
P135
P645
% Cells
ns
G1
S
G2/M
Cell Cycle
DMSO
Silmitasertib
G
Casp3/7 activity in normal colon PDOs
Caspase 3/7 (%)
DMSO
Silmitasertib 10 μM
Trametinib 30 nM
Combo
P134
P135
P645

Figure S5. Characterisation of normal colon PDOs and their response to silmitasertib and trametinib. (**A**) Representative images of haematoxylin and Eosin (H&E) staining and IHC staining for Ki67, MUC2, and CK20 in PDOs derived from normal mucosa tissue of patient P645. Organoids are shown in both undifferentiated (top row, cultured in IntestiCult OGM) and differentiated (bottom row, cultured in IntestiCult ODM) states. Insets highlight regions of interest. Ki67 positivity reduction indicates a lower proliferation rate after differentiation. MUC2 staining highlights mucin-producing cells, which are more pronounced in differentiated PDOs. CK20 staining is used to assess epithelial differentiation and indicates higher expression in differentiated PDOs. (**B**) Representative western blot images showing CKII-specific phosphosubstrate levels (pSerine/pThreonine), and β-actin loading control in mCRC PDOs and normal colon PDOs. (**C**) Quantification of CKII phosphosubstrate levels normalised to β-actin in mCRC PDOs compared to normal colon PDOs. For mCRC PDOs, each dot represents an individual PDO line (n=9); for normal colon PDOs, each dot represents an individual PDO line (n=3). Boxes indicate the median and interquartile range, and whiskers the minimum and maximum. Statistical significance was assessed using Mann-Whitney U test (**p=0.0091). (**D**) Western blot showing CKII-specific phosphosubstrate levels (pSer/pThr motif) and β-actin loading control in normal colon PDOs treated with silmitasertib 10 μM or DMSO (replicates 2 and 3 are shown). (**E**) Quantification of CKII phosphosubstrate levels before and after silmitasertib treatment (n=3 PDO lines; three independent biological replicates per line). Log-transformed OD ratios were analysed by two-way repeated-measures ANOVA with Šídák's multiple-comparison test within each line (ns=not significant, *p<0.05). (**F**) Bar plots showing percentage of cells in G1, S, and G2/M phases in the normal colon PDO lines P134 (left), P135 (middle) and P645 (right) before and after silmitasertib treatment. Data are presented as mean ± SD of three independent experiments. Statistical significance was determined by an unpaired t-test (ns=not significant). (**G**) Bar plots showing caspase-3/7 activity normalised to DMSO in P134, P135 and P645 across four treatment conditions. Bars represent the mean ± SD of three independent biological replicates per condition. Statistical significance was assessed by two-way ANOVA with Tukey's multiple-comparison test; no comparison reached significance.

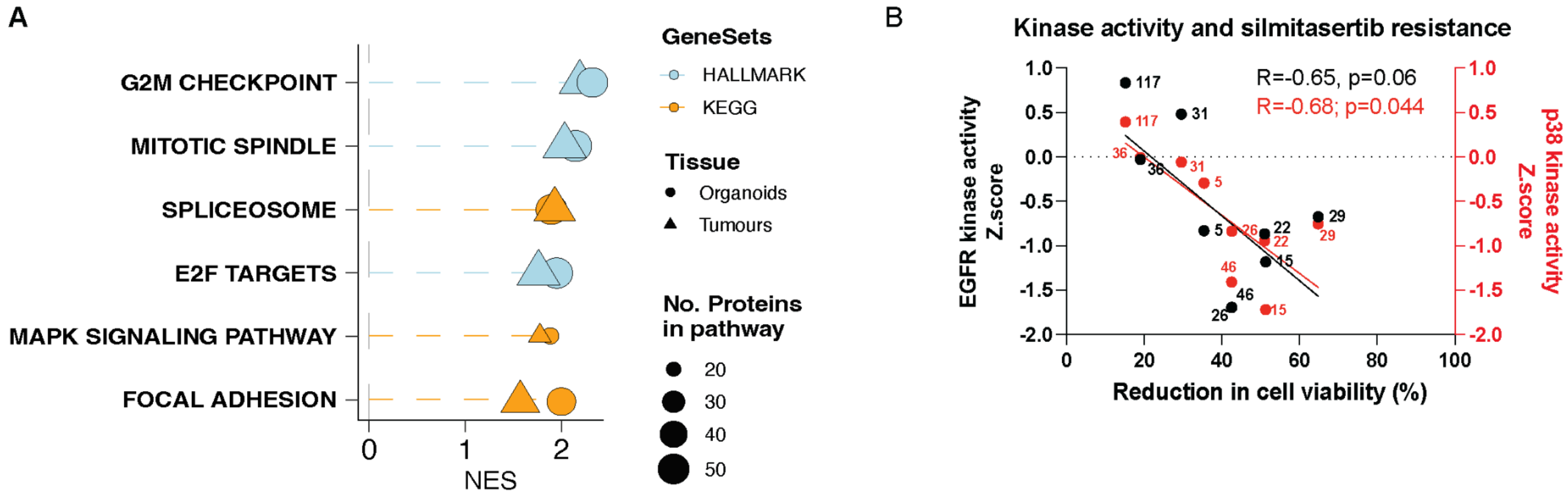

Figure S6. MAPK signalling is linked to silmitasertib antitumour efficacy. (**A**) Enrichment analysis of hallmark and KEGG gene sets based on detected phosphosubstrates. The normalised enrichment score (NES) is displayed on the x-axis. Triangles and circles represent the number of proteins in each pathway, with circles indicating PDOs and triangles indicating tumour tissues. The size of each symbol corresponds to the number of proteins in the pathway. (**B**) Correlation plot between kinase activity and silmitasertib's antitumour efficacy in PDOs. The kinase activity Z-scores for EGFR (black) and p38 (red) are plotted against silmitasertib-induced cell-killing activity across mCRC PDOs (n=9). The Pearson correlation coefficients are indicated: EGFR (R=−0.65, p=0.06), p38 (R=−0.68, p=0.044).

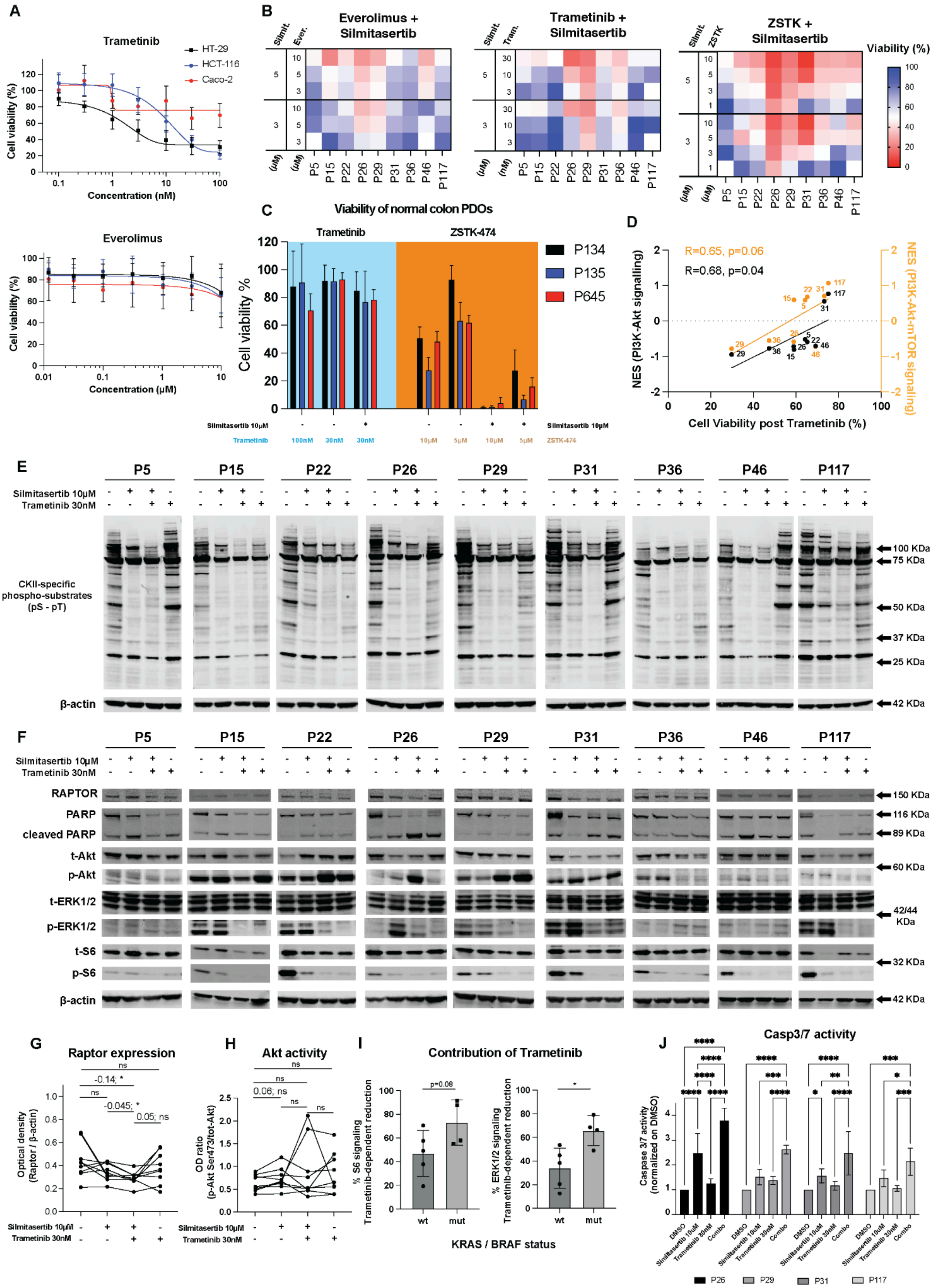

A
Trametinib
HT-29
HCT-116
Caco-2
Cell viability (%)
Concentration (nM)
Everolimus
Concentration (μM)
B
Everolimus + Silmitasertib
Trametinib + Silmitasertib
ZSTK + Silmitasertib
Silmit.
Ever.
Tram.
ZSTK
(μM)
P5
P15
P22
P26
P29
P31
P36
P46
P117
Viability (%)
C
Viability of normal colon PDOs
Trametinib
ZSTK-474
Cell viability %
P134
P135
P645
Silmitasertib 10μM
D
R=0.65, p=0.06
R=0.68, p=0.04
NES (PI3K-Akt signalling)
NES (PI3K-Akt-mTOR signaling)
Cell Viability post Trametinib (%)
E
Silmitasertib 10μM
Trametinib 30nM
CKII-specific phospho-substrates (pS - pT)
β-actin
100 KDa
75 KDa
50 KDa
37 KDa
25 KDa
42 KDa
F
RAPTOR
PARP
cleaved PARP
t-Akt
p-Akt
t-ERK1/2
p-ERK1/2
t-S6
p-S6
β-actin
150 KDa
116 KDa
89 KDa
60 KDa
42/44 KDa
32 KDa
42 KDa
G
Raptor expression
Optical density (Raptor / β-actin)
H
Akt activity
OD ratio (p-Akt Ser473/tot-Akt)
I
Contribution of Trametinib
% S6 signaling Trametinib-dependent reduction
% ERK1/2 signaling Trametinib-dependent reduction
KRAS / BRAF status
wt
mut
p=0.08
J
Casp3/7 activity
Caspase 3/7 activity (normalized on DMSO)
DMSO
Silmitasertib 10uM
Trametinib 30nM
Combo
P26
P29
P31
P117

Figure S7. Drug screening, normal colon organoid viability assessment, and molecular characterisation of silmitasertib and trametinib combination. (**A**) Dose-response curves showing the effect of trametinib (top) and everolimus (bottom) on cell viability in the selected CRC cell lines. As expected, trametinib exerted higher antitumour activity in *BRAF/KRAS*-mutant cell lines and limited effect on *wild-type* Caco2, while everolimus had overall a very modest effect at tested concentrations. Data are presented as mean ± SD of at least three independent experiments performed in triplicate. (**B**) Cell viability data in PDOs treated with combinations of silmitasertib with either everolimus (left), trametinib (centre), or ZSTK-474 (right) across increasing concentrations. These data complement data shown in **Figure 5B**. (**C**) Bar graphs showing the sensitivity of three normal colon PDO lines (P134, P135, and P645) upon trametinib, ZSTK-474 and their respective combination with silmitasertib 10 μM. In contrast to ZSTK-474, trametinib had little effect on normal colon PDO viability. Bars represent mean ± SD of at least three independent experiments. (**D**) Scatter plots showing positive correlations between PI3K-Akt (black, R=0.68, p=0.04) and PI3K-Akt-mTOR (orange, R=0.65, p=0.06) signalling NES scores and cell viability following trametinib treatment across mCRC PDOs (n=9). Pearson's correlation coefficient (R) and the corresponding p-value are indicated. (**E**, **F**) Representative western blot analysis for the measurement of CKII-specific phospho-substrates, PARP and MAPK and Akt-mTOR-S6 signalling following silmitasertib 10 μM and trametinib 30 nM, as a single agent or in combination, in the whole PDOs cohort. Band lanes were used to quantify CKII activity, as shown in **Figure 5E**. (**G**, **H**) Quantification of RAPTOR expression and Akt activity following single or co-treatment. Comparisons were performed using the paired Wilcoxon signed-rank test, with p-values adjusted for multiple comparisons using the Benjamini-Hochberg method across the five comparisons shown (ns=not significant, $*p<0.05$). (**I**) Bar graphs showing the contribution of trametinib to ERK1/2 and S6 signalling reduction in PDOs with either wild-type (wt) or mutant (mut) KRAS/BRAF status. The bars represent mean ± SD (wt, n=5; mut, n=4 PDO lines). Statistical significance was determined by an unpaired two-tailed t-test ($*p < 0.05$). (**J**) Bar plots showing caspase-3/7 activity normalised to DMSO in P26, P29, P31, and P117 across four treatment conditions. Bars represent the mean ± SD of three independent experiments. Statistical significance was determined by one-way ANOVA with Tukey's post-hoc test (*p<0.05, **p<0.01, ***p<0.001, ****p<0.0001).

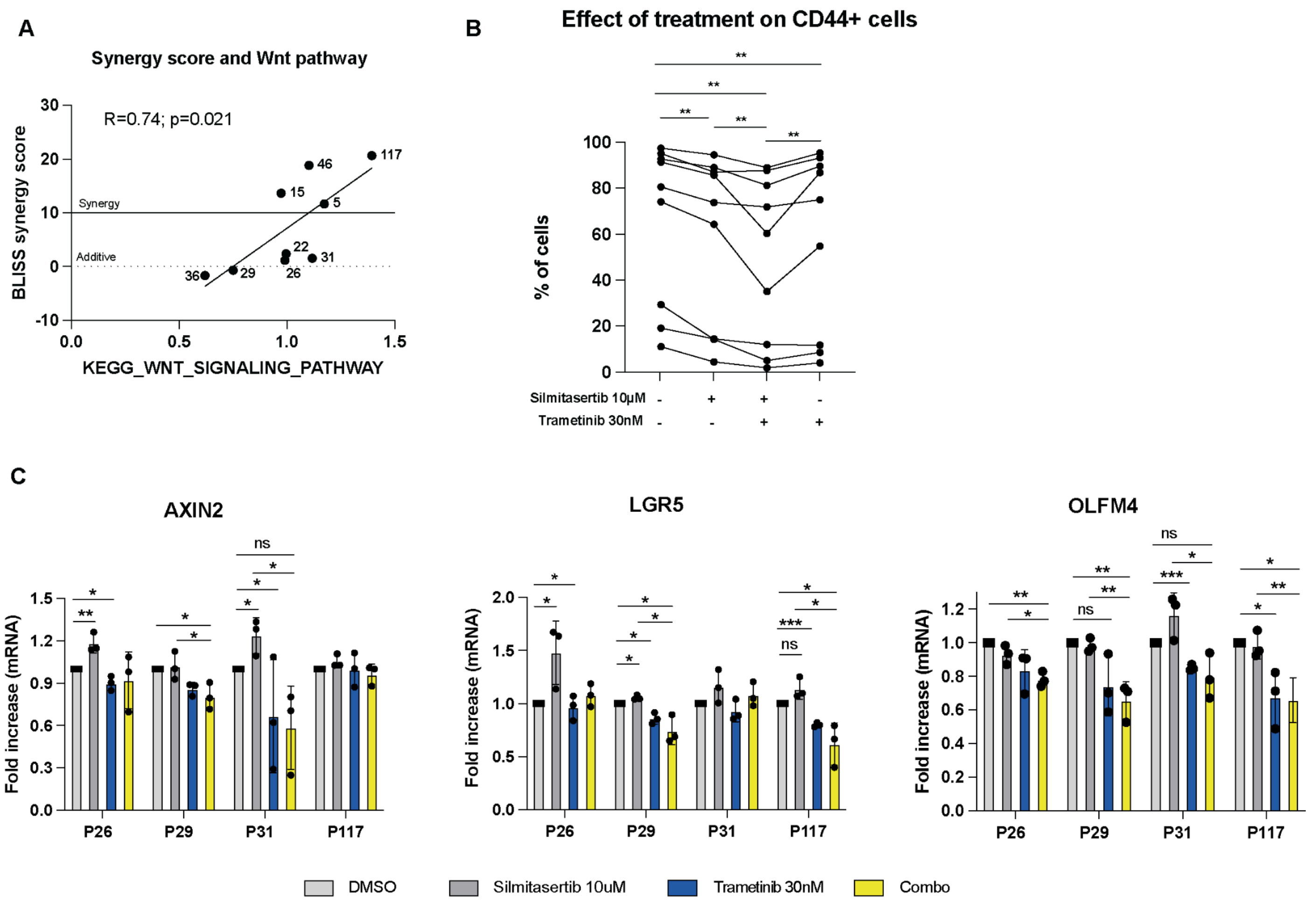

Figure S8. Effects of silmitasertib and trametinib on Wnt signalling and cancer stem cell markers. (**A**) Scatter plot showing the correlation between the synergy score calculated using the Bliss model and the KEGG WNT signalling pathway score across PDOs (n=9). Pearson's correlation coefficient and p-value are indicated (R=0.74, p=0.021). (**B**) Line graph showing the percentage of CD44+ cells in response to single or combined treatments across PDOs (n=9). Comparisons were performed using the paired Wilcoxon signed-rank test, with p-values adjusted for multiple comparisons using the Benjamini-Hochberg method across the five comparisons shown (**p<0.01). (**C**) Bar plots showing mRNA fold increase of the Wnt target gene *AXIN2* (left), and the cancer stem cell markers *LGR5* (middle) and *OLFM4* (right) after treatment in four mCRC PDOs (P26, P29, P31, P117). Conditions: DMSO (light grey), silmitasertib 10 µM (dark grey), trametinib 30 nM (blue), and combination (yellow). Data represent mean ± SD of three independent experiments. Statistical significance was determined by one-way ANOVA with a Tukey post-hoc test (*p<0.05, **p<0.01, ***p<0.001, ****p<0.0001).
