# Supplementary material for: Combined casein kinase II and MAPK signalling inhibition as a therapeutic strategy for metastatic colorectal cancer
Source: Exp Hematol Oncol. 2026 Nov 5;15:119. doi: 10.1186/s40164-026-00855-4 (PMC13737822; doi:10.1186/s40164-026-00855-4)
Supplement: Supplementary file 2 [file 40164_2026_855_MOESM1_ESM.docx]

**Additional File 1: Supplementary Methods**

**Combined Casein Kinase II and MAPK signalling inhibition as a therapeutic strategy for metastatic colorectal cancer**

**Marinucci et al.**

**Supplementary Methods**

**Patient cohort and tissue sample processing**

Clinical data were collected retrospectively, including patient age, sex and prior treatment (**Table 1**). Tissues were collected in the surgical theatre after resection and processed within 2 h. Prior to mechanical and enzymatic dissociation, mCRC tissue specimens were quickly washed in cold PBS (GIBCO, #10010023) plus 100 µg/ml primocin (InvivoGen, #ant-pm-05), and small pieces were cut and snap-frozen in liquid nitrogen for molecular profiling.

The Humanitas cohort yielded the PDO lines P563, P581 and P687 from liver metastases, lines P130 and P255 from primary colorectal tumours resected in treatment-naive patients, and the normal colon lines P134 and P135. The remaining validation lines from liver metastases (P145, P237 and P405) and the normal colon PDO line P645 were established from tissue collected at Clarunis, Basel, under approval no. 2019-02118.

**Patient-derived organoid generation and passaging**

Briefly, following washing with PBS plus primocin, tissues were minced into small pieces and subsequently enzymatically digested in 1 ml of advanced DMEM/F-12 FULL (GIBCO, #12634010) medium containing 2.5 mg/ml collagenase IV (Worthington, #LS004189), 0.1 mg/ml DNase IV (Sigma, #D5025), 20 μg/ml hyaluronidase V (Sigma, #H6254), 1% BSA (Sigma, #A3059), and 10 μM Y-27632 (Abmole Bioscience, #M1817) for 1 h at 37 °C under slow rotation and vigorous pipetting every 15 min. The tissue lysate was filtered through a 100 µm cell strainer, centrifuged at 300 g for 10 min, and then treated with Accutase (Sigma, #A6964) for 15 min at 37°C in order to dissociate the remaining fragments. After 5 min of centrifugation at 300 g, the cell pellet was suspended in PBS and cells were counted using trypan blue (GIBCO, #15250061), Countess™ Cell Counting Chamber Slides (Invitrogen, #C10228), and Countess™ II FL Automated Cell Counter (Invitrogen, #AMQAF1000). Then, cells were resuspended in growth factor reduced Matrigel (Corning, #356231) and seeded as drops in a tissue-culture plate. After polymerisation of the Matrigel, a CRC-specific growing medium was added to the cells. The CRC-specific PDOs medium was slightly modified compared to previously published protocols [[1]](https://paperpile.com/c/PKOHry/tj4S) and was composed of Advanced DMEM/F12 implemented with 1x B27 supplement (ThermoFisher, #17504044), 10 mM Nicotinamide (Sigma-Aldrich, #N0636), 1.25mM N-acetyl-L-cysteine (Sigma-Aldrich, #A9265-25G), 500nM A83-01 (PeproTech, #2939), 50ng/ml EGF (PeproTech, #AF-100-15), 100 ng/ml Noggin (PeproTech, #120-10C), 500 ng/ml R-spondin1 (R&D Systems, #4645-RS-100), 10nM PGE2 (Tocris, #2296), 10 µM SB202190 (Sigma-Aldrich, #S7076), 10 µM Y-27632 (Abmole Bioscience, #M1817), 10 nM Gastrin (Sigma-Aldrich, #G9145), 100 µg/ml primocin. Organoid medium was changed every 2-3 days, and, when needed, organoids were passaged after dissociation with TrypLE™ Express Enzyme (1X) (GIBCO, #12605-010).

A similar protocol was employed for the generation of PDOs from normal colon tissues, with little modification. After washing, colon tissues were cut in 1-2mm (max) pieces to prevent crypt disruption. Enzymatic digestion solution was further implemented with 5 mM EDTA (Gibco, #15575020) and incubation time was shortened to a maximum of 30 min. Normal colon PDOs were passaged and maintained using IntestiCult™ Organoid Growth Medium (Stemcell technologies, #06010) and differentiated using IntestiCult™ Organoid Differentiation Medium (Stemcell technologies, #100-0214) as described by the manufacturer. Only differentiated normal colon PDOs were used for drug treatment and molecular biology experiments.

Organoid medium was changed every 2-3 days, and, when needed, organoids were passaged after dissociation with Gentle Dissociation Reagent (1X) (Stemcell technologies, #100-0485, 100-1077).

All PDOs were maintained in long-term cultures, except for the P17 line, whose growth rate decreased over time and therefore it was not included in whole-exome sequencing, drug screenings or functional experiments, an inconvenience already observed in previous works [[2]](https://paperpile.com/c/PKOHry/8Hzn2).

**Cell lines maintenance**

CRC cell lines HT-29, CaCo2 and HCT-116 were monitored regularly for mycoplasma contamination by PCR using specific primers as described previously [[3]](https://paperpile.com/c/PKOHry/UzlpT). Cells were cultured in DMEM (GIBCO, #11965092) supplemented with 10% foetal bovine serum (GIBCO, #A5256801), 1X non-essential amino acids (GIBCO, #11140050) and 100 U/mL penicillin/streptomycin (GIBCO, #15140122). Cells were incubated at 37 °C in a humidified atmosphere with 5% CO₂ and passaged at 80% confluence using 0.05% trypsin-EDTA (GIBCO, #25300054). Exponentially growing cells were used for all experiments.

**Immunohistochemistry**

Tissue and PDO sections were rehydrated and immunohistochemical staining was performed on a Benchmark immunohistochemistry staining system, VENTANA BenchMark Special Stains system (Roche). The antibodies used are listed in **Additional file 2.** Images were acquired using an Olympus BX46 microscope and evaluated independently by two experienced pathologists (MC, LMT).

**Nucleic acid extraction and RT-qPCR**

Samples were lysed in DNA/RNA Lysis Buffer and lysates were transferred to a Zymo-Spin column to bind DNA; the flow-through containing RNA was transferred to a new column, treated with DNase I, and both nucleic acids were washed and eluted in water. RNA was reverse-transcribed using the High-Capacity cDNA Reverse Transcription kit (Applied Biosystems, #4368813). RT-qPCR was performed by amplifying 10 ng cDNA with PowerUp SYBR Green Master Mix (Applied Biosystems, #A25742) and the primers listed in **Additional file 2**, according to the manufacturer's instructions.

**Whole-exome sequencing**

Sequence reads were aligned to the reference human genome GRCh38 using Burrows-Wheeler Aligner (BWA [[4]](https://paperpile.com/c/PKOHry/htLts), v.0.7.17). Local realignment, duplicate removal and base quality adjustment were performed using the Genome Analysis Toolkit (GATK [[5]](https://paperpile.com/c/PKOHry/mB70), v. 3.6) and Picard (<http://broadinstitute.github.io/picard/>, v.2.20.0).

Somatic single nucleotide variants (SNVs) and small insertions and deletions (indels) were detected using Mutect2 [[6]](https://paperpile.com/c/PKOHry/g5Syc) (GATK v.4.2.4.1) and Strelka2 [[7]](https://paperpile.com/c/PKOHry/qI70) v.2.9.10. We filtered out SNVs and indels outside of the target regions and/or those supported by <3 reads and/or those where the total read coverage was below 10 in the tumour sample or 5 in the normal sample. We excluded variants for which the tumour VAF was <5 times that of the paired non-tumour VAF. We further excluded variants identified in a panel of 210 non-tumour samples captured and sequenced using the same protocols and created using Mutect2 and CreateSomaticPanelOfNormals from GATK v.4.1.4.1. Mutations affecting hotspot residues were annotated (<https://github.com/charlottekyng/cancer_hotspots>). To account for the possibility that a somatic variant found in a sample is also present in another sample from the same donor but below the detection threshold or failing the above-mentioned read coverage filters, we re-called such variants using HaplotypeCaller (GATK v.4.1.3.0) using the options --genotyping-mode GENOTYPE_GIVEN_ALLELES --output-mode EMIT_ALL_SITES. In this case, variants supported by at least two alternative reads were considered present.

**Proteomic and phosphoproteomic profiling and analysis**

Proteomic and phosphoproteomic profiling were performed on 10 CRC tissues and matched PDOs, as well as 6 normal colon tissues. For the CRC and normal tissues, three pieces from the same resection were used. Three biological replicates were used for the PDOs. PDOs were lysed by sonication in 2M guanidine hydrochloride, 100mM ammonium bicarbonate, 5mM TCEP, and phosphatase inhibitors (Sigma, #P5726 & P0044). Proteins were subsequently heat-reduced and alkylated with 10 mM chloroacetamide. Samples were then diluted with 100 mM ammonium bicarbonate to a final guanidine hydrochloride concentration of 0.5 M and digested by incubation with sequencing-grade modified trypsin (1/50, w/w; Promega, #V5111) for 12 h at 37°C. After digestion, samples were acidified with 5% TFA and peptides were purified using C18 reverse-phase spin columns (Macrospin, Harvard Apparatus, #74-7242), dried under vacuum and stored at -20°C until further use. Peptide samples were enriched for phosphorylated peptides using Fe(III)-IMAC cartridges on an AssayMAP Bravo platform as described [[8]](https://paperpile.com/c/PKOHry/ewJzd).

Phospho-enriched peptides were resuspended in 0.1% aqueous formic acid and subjected to LC-MS/MS analysis using an Orbitrap Fusion Lumos Mass Spectrometer fitted with an EASY-nLC 1200 (both Thermo Fisher Scientific) and a column heater set to 60°C. Peptides were resolved using an RP-HPLC column (75μm × 36cm) packed in-house with C18 resin (ReproSil-Pur C18–AQ, 1.9 μm resin; Dr. Maisch GmbH, #r119.aq.). Peptides were fragmented by HCD (Higher-energy collisional dissociation).

The acquired raw files were imported into the Progenesis QI software (v2.0, Nonlinear Dynamics Limited), which was used to extract peptide precursor ion intensities across all samples. The generated mgf file was searched using MASCOT against a human database. Quantitative analysis results from label-free quantification were normalised and statistically analysed using SafeQuant R package v.2.3.2[[9]](https://paperpile.com/c/PKOHry/hXcyw)

We obtained data for 6275 proteins that were quantified in at least one run in all samples. The raw intensities (combined intensity of the peptides of the protein) of the triplicates were averaged, and the mean values were transformed by the logarithm to the base 2. Next, the values were normalised by median subtraction. We removed proteins only identified by a modification site and proteins that matched the reversed part of the decoy database or commonly occurring contaminants. Then we removed proteins with >50% missing values in ≥2 groups (tumour tissue, PDOs, or normal colon), resulting in 4254 proteins for further analysis.

The phosphosite data were averaged and normalised in the same manner as the proteomics data described above. After filtering out potential contaminants, we retained 6964 phosphosites for downstream analysis. This number was obtained after removing phosphosites with low localisation confidence and those with more than 50% missing values in at least two groups (normal colon, tumour tissue or PDOs).

**Data analysis**

*Proteomics and Phosphoproteomics*

Consensus hierarchical clustering was performed using the 500 most variable features on proteomics using the ConsensusClusterPlus v1.5.8 [[10]](https://paperpile.com/c/PKOHry/sioW0), with the following parameters: maxK = 6, reps = 100 bootstraps, clusterAlg = ward.d2, distance = pearson. The number of clusters was determined by the delta plot of the relative change in the area under the cumulative distribution function curve and the average silhouette distance for consensus clusters. KEGG enrichment analysis was performed to functionally annotate protein clusters using rbioapi v0.8.1 [[11]](https://paperpile.com/c/PKOHry/wPIrV).

To obtain a global picture of protein and phosphosite abundance for the tumour tissue and PDOs versus healthy colon tissues, we fit a probabilistic dropout model and tested for differentially abundant proteins and phosphosites on the log2-transformed intensities using proDA v1.8 [[12]](https://paperpile.com/c/PKOHry/whSm). Proteins or phosphorylation sites with adjusted p < 0.05 were considered differentially expressed. Pathway analysis for the proteomics data on the resultant t statistics was performed by gene set enrichment analysis (GSEA) using the fgsea package v1.2 [[13]](https://paperpile.com/c/PKOHry/yyo6m) and KEGG, Hallmarks and Reactome gene sets derived from msigdbr v7.5.1 [[14]](https://paperpile.com/c/PKOHry/0cfaW).

To estimate changes in kinase activities, we performed kinase-substrate enrichment analysis (KSEA) using the KSEAapp R package v0.99 [[15, 16]](https://paperpile.com/c/PKOHry/JKwkr+ceJk) using NetworKIN.cutoff = 5, the log fold-change and p-values computed from differential abundance analysis of both the phosphorylation site levels and phosphorylation site levels normalised by overall protein levels as input. Known and predicted kinase-substrate site relationships from PhosphoSitePlus® [[17]](https://paperpile.com/c/PKOHry/dBKek) (PSP) or NetworKIN [[18]](https://paperpile.com/c/PKOHry/ciYpX) 3.0 were used as the Kinase-Substrate sources. Kinases with Benjamini–Hochberg-corrected FDR <0.05 were considered significantly enriched.

*Statistical analysis*: For (phospho)proteomics data, values imputed by using the 'Missing not At Random' (MNAR) method, which uses random draws from a Gaussian distribution left-shifted by 1.8 StDev and batch-corrected for tissue type by limma [[19]](https://paperpile.com/c/PKOHry/9phOx) were used to remove the effect of missing values and systemic effects introduced by organoid growth for use in principal component analysis (PCA). PCA of proteome and phosphoproteome data was performed using ‘prcomp’ from the stats base R library, where the top 500 most variable log2 median-normalised batch-corrected abundance values were used. Overall immune cell gene scores were computed on the normalised proteomics data using the ConsensusTME v0.0.1.9 [[20]](https://paperpile.com/c/PKOHry/o2hZC).

**Drug treatment on CRC PDO and cell lines**

Cells were seeded in 384 well plates (Greiner, #781986) and grown for 24h. Drugs were then added to the medium with a D300e Digital Dispenser (Tecan) and, after 72h, cell viability was measured using CellTiter-Glo (Promega, #G7570), according to manufacturer instructions, on the Varioskan LUX multifunctional microplate reader (Thermo Scientific). CRC PDOs were seeded in a 96-well plate (TTP, #92096) at a density of 7 × 10³ cells in 7 µL matrigel drop. After 48 h, when organoids started to be clearly visible, CRC-specific culture medium was replenished with a fresh one containing drugs and viability was measured after 4 days using CellTiter-Glo 3D reagent (Promega, G9683). Results were normalised to vehicle-containing medium (DMSO 0.1%). Drug interaction was computed via the use of the SynergyFinder ([https://synergyfinder.fimm.fi](https://synergyfinder.fimm.fi/))[[21]](https://paperpile.com/c/PKOHry/T7Mpu), version 3.0, using the highest single agent (HSA), Bliss [[22]](https://paperpile.com/c/PKOHry/YF0ZH), Loewe, and zero interaction potency (ZIP) reference models. The final scores were classified as synergistic (synergy score > 10), additive (-10 < synergy score ≤ 10) or antagonistic (synergy score < -10) with the Bliss score.

**Immunoblotting**

Total proteins were extracted from both tissues and PDOs by using a Co-IP lysis buffer (100 mmol/L NaCl, 50 mmol/L Tris pH 7.5, 1 mmol/L EDTA, 0.1% Triton X-100) supplemented with 1× protease inhibitors (cOMPLETE, catalogue number 4693132001, Roche) and 1× phosphatase inhibitors (PhosSTOP, catalog number 4906845001, Roche). Cell lysates were then treated with 1× reducing agent, 1× loading buffer, boiled 5 min at 95 °C and loaded onto neutral pH, pre-cast, discontinuous SDS-PAGE mini-gel system[[23]](https://paperpile.com/c/PKOHry/WDzci). After electrophoresis, proteins were transferred to nitrocellulose membranes using the Trans-Blot Turbo Transfer System (Bio-Rad). The trans-blotted membranes were blocked for 1 h in TBS-T 0.1%, 5% milk and then probed with appropriate primary antibodies overnight at 4 °C, except the anti-β-actin primary antibody that was probed for 1h at RT. Next, the membranes were incubated for 1 h at room temperature with fluorescent secondary goat anti-mouse (IRDye 680) or anti-rabbit (IRDye 800) antibodies (both from LI-COR Biosciences). Employed primary and secondary antibodies are listed in **Additional file 2**. Blots were scanned using the Odyssey Infrared Imaging System (LI-COR Biosciences) and band intensity was quantified using ImageJ software. The signal from the protein of interest was normalised on a loading control band (β-actin or GAPDH). All experiments were performed and analysed at least in triplicate; lanes affected by technical artefacts were excluded from quantification. “Protein activity” was calculated by normalising the levels of phosphorylated protein on the respective total protein amount, reflecting the approach used to analyse the phosphoproteomic dataset.

**Flow cytometry analysis**

Cell cycle was assessed via the DAPI-mediated detection of cellular DNA content through flow cytometry analysis to identify the proportion of cells that are in G1, S or G2/M phase [[23]](https://paperpile.com/c/PKOHry/WDzci). PDOs were grown for 48h and treated with silmitasertib 10 µM or 0.1% vehicle DMSO for 24 h. At the end of the treatment, PDOs were treated with trypsin-EDTA 0.25% (Gibco) to obtain a single cell suspension and cells were fixed with cold 70% ethanol for 30 min. After washing and careful removal of the ethanol, DNA staining was performed with a PBS-based solution with Tween-20 0.1% and DAPI 0.1 µg/ml (BioLegend, #422801). Cells were washed and resuspended in a FACS buffer (2 mM EDTA and 0.5% BSA) and DAPI fluorescence was detected by flow cytometry (CytoFLEX, Beckman Coulter). Data analysis was performed using FlowJo 10.10.0 (BD, USA).

CRC cancer stem cell markers CD44 and CD133 were assessed via flow cytometry. PDOs were seeded and grown for 48h and then treated for 24h with vehicle (DMSO), silmitasertib 10µM, trametinib 30nM or their combination. Matrigel drops containing PDOs were harvested and disrupted with cold PBS. After 5 min incubation in ice, PDOs were centrifuged at 4 °C for 5 min and resuspended with TrypLE Express (Gibco). After 4 min incubation at 37 °C, PDOs were mechanically disrupted by using a 25G needle and enzymatic digestion was stopped by adding DMEM containing 10% FBS. After 2 washing steps, cells were stained for 20 min at 4 °C with FACS buffer containing APC-conjugated anti-CD133, and AF488-conjugated anti-CD44 at a final cell density of around 1 × 10⁶ cells/ml (**Additional file 2**). After 2 washing steps, fluorescence signals were detected with CytoFLEX flow cytometer (Beckman Coulter). Flow cytometry data were analysed with FlowJo 10.10.0 (BD).

**Caspase-3/7 activity**

PDOs were seeded in a 96-well plate (TTP, #92096) at 7×10³ cells in 7 µL Matrigel domes. After 48 h, when organoids were clearly visible, the culture medium was replenished with fresh medium containing drugs. Caspase-3/7 activity was measured after 2 days using the Caspase-Glo 3/7 3D assay (Promega, #G8981) on a Varioskan multifunctional microplate reader (Thermo Fisher) with SkanIt Software 6.0.2. Results were normalised to vehicle-containing medium (0.1 % DMSO).

**Supplementary References**

[1. Sato T, Stange DE, Ferrante M, Vries RGJ, Van Es JH, Van den Brink S, et al. Long-term expansion of epithelial organoids from human colon, adenoma, adenocarcinoma, and Barrett’s epithelium. Gastroenterology. 2011;141:1762–72. https://doi.org/10.1053/j.gastro.2011.07.050.](http://paperpile.com/b/PKOHry/tj4S)

[2. van de Wetering M, Francies HE, Francis JM, Bounova G, Iorio F, Pronk A, et al. Prospective derivation of a living organoid biobank of colorectal cancer patients. Cell. 2015;161:933–45. https://doi.org/10.1016/j.cell.2015.03.053.](http://paperpile.com/b/PKOHry/8Hzn2)

[3. Marinucci M, Ercan C, Taha-Mehlitz S, Fourie L, Panebianco F, Bianco G, et al. Standardizing Patient-Derived Organoid Generation Workflow to Avoid Microbial Contamination From Colorectal Cancer Tissues. Front Oncol. 2021;11:781833. https://doi.org/10.3389/fonc.2021.781833.](http://paperpile.com/b/PKOHry/UzlpT)

[4. Li H, Durbin R. Fast and accurate short read alignment with Burrows-Wheeler transform. Bioinformatics. 2009;25:1754–60. https://doi.org/10.1093/bioinformatics/btp324.](http://paperpile.com/b/PKOHry/htLts)

[5. McKenna A, Hanna M, Banks E, Sivachenko A, Cibulskis K, Kernytsky A, et al. The Genome Analysis Toolkit: a MapReduce framework for analyzing next-generation DNA sequencing data. Genome Res. 2010;20:1297–303. https://doi.org/10.1101/gr.107524.110.](http://paperpile.com/b/PKOHry/mB70)

[6. Cibulskis K, Lawrence MS, Carter SL, Sivachenko A, Jaffe D, Sougnez C, et al. Sensitive detection of somatic point mutations in impure and heterogeneous cancer samples. Nat Biotechnol. 2013;31:213–9. https://doi.org/10.1038/nbt.2514.](http://paperpile.com/b/PKOHry/g5Syc)

[7. Kim S, Scheffler K, Halpern AL, Bekritsky MA, Noh E, Källberg M, et al. Strelka2: fast and accurate calling of germline and somatic variants. Nat Methods. 2018;15:591–4. https://doi.org/10.1038/s41592-018-0051-x.](http://paperpile.com/b/PKOHry/qI70)

[8. Post H, Penning R, Fitzpatrick MA, Garrigues LB, Wu W, MacGillavry HD, et al. Robust, Sensitive, and Automated Phosphopeptide Enrichment Optimized for Low Sample Amounts Applied to Primary Hippocampal Neurons. J Proteome Res. 2017;16:728–37. https://doi.org/10.1021/acs.jproteome.6b00753.](http://paperpile.com/b/PKOHry/ewJzd)

[9. Ahrné E, Glatter T, Viganò C, Schubert C von, Nigg EA, Schmidt A. Evaluation and Improvement of Quantification Accuracy in Isobaric Mass Tag-Based Protein Quantification Experiments. J Proteome Res. 2016;15:2537–47. https://doi.org/10.1021/acs.jproteome.6b00066.](http://paperpile.com/b/PKOHry/hXcyw)

[10. Wilkerson MD, Hayes DN. ConsensusClusterPlus: a class discovery tool with confidence assessments and item tracking. Bioinformatics. 2010;26:1572–3. https://doi.org/10.1093/bioinformatics/btq170.](http://paperpile.com/b/PKOHry/sioW0)

[11. Rezwani M, Pourfathollah AA, Noorbakhsh F. rbioapi: user-friendly R interface to biologic web services’ API. Bioinformatics. 2022;38:2952–3. https://doi.org/10.1093/bioinformatics/btac172.](http://paperpile.com/b/PKOHry/wPIrV)

[12. Ahlmann-Eltze C, Anders S. ProDA: Probabilistic dropout analysis for identifying differentially abundant proteins in label-free mass spectrometry. bioRxiv. 2019. https://doi.org/10.1101/661496.](http://paperpile.com/b/PKOHry/whSm)

[13. Korotkevich G, Sukhov V, Budin N, Shpak B, Artyomov MN, Sergushichev A. Fast gene set enrichment analysis. bioRxiv. 2016. https://doi.org/10.1101/060012.](http://paperpile.com/b/PKOHry/yyo6m)

[14. Dolgalev I. MSigDB Gene Sets for Multiple Organisms in a Tidy Data Format [R package msigdbr version 7.5.1]. 2022.](http://paperpile.com/b/PKOHry/0cfaW)

[15. Casado P, Rodriguez-Prados J-C, Cosulich SC, Guichard S, Vanhaesebroeck B, Joel S, et al. Kinase-substrate enrichment analysis provides insights into the heterogeneity of signaling pathway activation in leukemia cells. Sci Signal. 2013;6:rs6. https://doi.org/10.1126/scisignal.2003573.](http://paperpile.com/b/PKOHry/JKwkr)

[16. Wiredja DD, Koyutürk M, Chance MR. The KSEA App: a web-based tool for kinase activity inference from quantitative phosphoproteomics. Bioinformatics. 2017;33:3489–91. https://doi.org/10.1093/bioinformatics/btx415.](http://paperpile.com/b/PKOHry/ceJk)

[17. Hornbeck PV, Zhang B, Murray B, Kornhauser JM, Latham V, Skrzypek E. PhosphoSitePlus, 2014: mutations, PTMs and recalibrations. Nucleic Acids Res. 2015;43 Database issue:D512–20. https://doi.org/10.1093/nar/gku1267.](http://paperpile.com/b/PKOHry/dBKek)

[18. Horn H, Schoof EM, Kim J, Robin X, Miller ML, Diella F, et al. KinomeXplorer: an integrated platform for kinome biology studies. Nat Methods. 2014;11:603–4. https://doi.org/10.1038/nmeth.2968.](http://paperpile.com/b/PKOHry/ciYpX)

[19. Ritchie ME, Phipson B, Wu D, Hu Y, Law CW, Shi W, et al. limma powers differential expression analyses for RNA-sequencing and microarray studies. Nucleic Acids Res. 2015;43:e47. https://doi.org/10.1093/nar/gkv007.](http://paperpile.com/b/PKOHry/9phOx)

[20. Jiménez-Sánchez A, Cast O, Miller ML. Comprehensive Benchmarking and Integration of Tumor Microenvironment Cell Estimation Methods. Cancer Res. 2019;79:6238–46. https://doi.org/10.1158/0008-5472.CAN-18-3560.](http://paperpile.com/b/PKOHry/o2hZC)

[21. Ianevski A, Giri AK, Aittokallio T. SynergyFinder 3.0: an interactive analysis and consensus interpretation of multi-drug synergies across multiple samples. Nucleic Acids Res. 2022;50:W739–43. https://doi.org/10.1093/nar/gkac382.](http://paperpile.com/b/PKOHry/T7Mpu)

[22. Liu Q, Yin X, Languino LR, Altieri DC. Evaluation of drug combination effect using a Bliss independence dose-response surface model. Stat Biopharm Res. 2018;10:112–22. https://doi.org/10.1080/19466315.2018.1437071.](http://paperpile.com/b/PKOHry/YF0ZH)

[23. Taha-Mehlitz S, Bianco G, Coto-Llerena M, Kancherla V, Bantug GR, Gallon J, et al. Adenylosuccinate lyase is oncogenic in colorectal cancer by causing mitochondrial dysfunction and independent activation of NRF2 and mTOR-MYC-axis. Theranostics. 2021;11:4011–29. https://doi.org/10.7150/thno.50051.](http://paperpile.com/b/PKOHry/WDzci)
